# Demographic history shaped geographical patterns of deleterious mutation load in a broadly distributed Pacific Salmon

**DOI:** 10.1371/journal.pgen.1008348

**Published:** 2020-08-26

**Authors:** Quentin Rougemont, Jean-Sébastien Moore, Thibault Leroy, Eric Normandeau, Eric B. Rondeau, Ruth E. Withler, Donald M. Van Doornik, Penelope A. Crane, Kerry A. Naish, John Carlos Garza, Terry D. Beacham, Ben F. Koop, Louis Bernatchez

**Affiliations:** 1 Département de Biologie, Institut de Biologie Intégrative et des Systèmes (IBIS), Université Laval, Québec, Québec, Canada; 2 ISEM, Univ. Montpellier, CNRS, EPHE, IRD, Montpellier, France; 3 Department of Botany & Biodiversity Research, University of Vienna, Vienna, Austria; 4 Centre for Biomedical Research, University of Victoria, Victoria, BC, Canada; 5 Department of Biology, University of Victoria, Victoria, BC, Canada; 6 Department of Fisheries and Ocean, Pacific Biological Station, Nanaimo, British Columbia, Canada; 7 National Oceanic and Atmospheric Administration, National Marine Fisheries Service, Northwest Fisheries Science Center, Manchester Research Station, Port Orchard, Washington, United States of America; 8 Conservation Genetics Laboratory, U.S. Fish and Wildlife Service, Anchorage, Alaska, United States of America; 9 School of Aquatic and Fishery Sciences, University of Washington, Seattle, WA, United States of America; 10 Fisheries Ecology Division, Southwest Fisheries Science Center, National Marine Fisheries Service and Institute of Marine Sciences, University of California–Santa Cruz, Santa Cruz, California, United States of America; University of Wyoming, UNITED STATES

## Abstract

A thorough reconstruction of historical processes is essential for a comprehensive understanding of the mechanisms shaping patterns of genetic diversity. Indeed, past and current conditions influencing effective population size have important evolutionary implications for the efficacy of selection, increased accumulation of deleterious mutations, and loss of adaptive potential. Here, we gather extensive genome-wide data that represent the extant diversity of the Coho salmon (*Oncorhynchus kisutch*) to address two objectives. We demonstrate that a single glacial refugium is the source of most of the present-day genetic diversity, with detectable inputs from a putative secondary micro-refugium. We found statistical support for a scenario whereby ancestral populations located south of the ice sheets expanded recently, swamping out most of the diversity from other putative micro-refugia. Demographic inferences revealed that genetic diversity was also affected by linked selection in large parts of the genome. Moreover, we demonstrate that the recent demographic history of this species generated regional differences in the load of deleterious mutations among populations, a finding that mirrors recent results from human populations and provides increased support for models of expansion load. We propose that insights from these historical inferences should be better integrated in conservation planning of wild organisms, which currently focuses largely on neutral genetic diversity and local adaptation, with the role of potentially maladaptive variation being generally ignored.

## Author summary

Reconstruction of a species’ past demographic history from genetic data can highlight historical factors that have shaped the distribution of genetic diversity along its genome and its geographic range. Here, we combine genotyping-by-sequencing with demographic modelling to address these issues in the Coho salmon, a Pacific salmon of conservation concern in some parts of its range, notably in the south. Our demographic reconstructions reveal a linear decrease in genetic diversity toward the north of the species range, supporting the hypothesis of a northern route of postglacial recolonization from a single major southern refugium. As predicted by theory, we also observed a higher proportion of deleterious mutations in the most distant populations from this refugium. Beyond this general pattern, among-site variation in the proportion of deleterious mutations is consistent with different local trends in effective population sizes. Our results highlight the potential importance of understanding historical factors that have shaped geographic patterns of the distribution of deleterious mutations in order to implement effective management programs for the conservation of wild populations. Such fundamental knowledge of human historical demography is now having major impacts on health sciences, and we argue it is time to integrate such approaches in conservation science as well.

## Introduction

Historical climate variation has had a major influence on the current distribution of species genetic diversity [1]. The Pleistocene glaciations, in particular, resulted in major contractions in the geographical distributions of many species into refugia that persisted in unglaciated areas [2–4]. Postglacial range expansions often led to contact between ancestral populations previously segregated in different refugia [2,4]. The effects of long-term climate change combined with recent human-induced population declines in wild animal populations can foster genetic changes including a loss of genetic diversity, increased inbreeding, increased load of deleterious mutations, and a loss of local adaptation [5].

In this context, understanding the impacts of historical climatic oscillations on the demographic history of a given species is crucial. Namely, it is important to understand how historical demographic events influenced the present-day geographic distribution of the within-species genetic diversity across its range. Here it is noteworthy that while the majority of studies on wild populations have focused on patterns of neutral and potentially adaptive genetic diversity, the importance of potentially maladaptive variation (e.g. patterns of deleterious mutation load) has generally been ignored. By disentangling past and current drivers of range-wide genomic diversity, this information can inform management and conservation decisions [6]. Beyond conservation implications, such context provides a unique opportunity to address outstanding questions in evolutionary biology: How is the efficacy of selection affected by historical processes that resulted in population expansion or bottleneck [7,8]? What are the demographic conditions required to generate substantial differences in deleterious load among populations [7–9]?

A major challenge in understanding drivers of genome-wide patterns of diversity is that different demographic processes can lead to similar contemporary genomic footprints [10]. As populations diverge, they accumulate genetic incompatibilities forming barriers to gene flow [11], while the rest of the genome may continue to be freely exchanged. Consequently, the genomic landscape of divergence is expected to vary, with greater differences along the genome at genomic barriers as compared to genomic regions exhibiting ongoing gene flow. However, similar patterns of heterogeneous genome-wide divergence can also be due to genetic hitchhiking of neutral alleles linked to selective sweeps [12,13] or to background selection (BGS;[12,14,15]]). These two processes, collectively referred to as linked selection, result in a reduction in nucleotide diversity in the vicinity of the sites targeted by positive or purifying selection. Due to its diversity-reducing effect, linked selection can be modeled as a local reduction of effective population size (*Ne)* [15]. It is now recognized that neglecting linked selection can bias demographic inferences [16,17] or lead to false adaptive interpretations [18]. Inference methods that incorporate variation in local effective population size and migration rate can help better understand how demography unfolds through time.

An understanding of historical demography is also essential for a sound interpretation of patterns of deleterious mutation load observed among contemporary populations [7,9]. Population bottlenecks are predicted to reduce potential for local adaptation, but also to reduce standing genetic variation and the efficacy of selection [8,19]. In turn, a reduced efficacy of purifying selection leads to an increase in the number of deleterious variants segregating in a population, thus reducing its fitness, and can result in maladaptation and/or reduced adaptive potential in bottlenecked populations. From a conservation standpoint, populations harboring an elevated number of deleterious variants might need to be monitored more closely to minimize extinction risks [20].

Combining population genomics data with demographic modelling represents a powerful strategy to test alternative hypotheses about historical drivers of existing genomic diversity. Previous studies employing a similar approach have focused mostly on species with a narrow geographic range, such as those inhabiting small islands [21,22], on the verge of extinction [23–26], or that experienced a strong bottleneck [27]. Few studies, however, have used demographic modelling to understand how historical processes have shaped the geographical patterns in the distribution of genomic diversity in a more broadly distributed species. An exception to this observation is the vast literature on demographic reconstructions of human populations. Long-lasting debates in this literature regarding the role of demography in generating mutation load differences among populations [9,28,29] could benefit from studies of species displaying similarly complex demographic histories and broad geographic distributions.

Salmonid fishes are economically important species that have suffered recent demographic declines [30,31]. This is particularly the case for Coho salmon (*Oncorhynchus kisutch*), one of the five anadromous species of Pacific salmon that supports important recreational and indigenous subsistence fisheries, and which has suffered dramatic population declines (> 90%) over the last three decades in parts of its range [32]. A previous study investigated the range-wide population structure and demographic history of the species and found a cline of decreasing diversity from south to north, as well as some endemic diversity in small putative refugia [33] (see also [34]). This study indicated that Coho salmon may have survived the last glacial maximum (LGM, i.e. the Fraser Glaciation in British Columbia, and the McConnell/McCauley Glaciation in Yukon and Alaska; 23 to 18 Ky ago) in unglaciated areas of Haida Gwaii and Beringia in addition to areas south of the ice sheets. This study, however, predates the genomic era and could not eliminate alternative hypotheses regarding the origin and number of glacial refugia during the LGM. In North America, the species is currently distributed from California to Alaska [35]. Unglaciated areas that could potentially serve as glacial refugia persisted both north (e.g. the Beringian refugium in Alaska, the Yukon Territory of Canada and areas of Asia and the Bering Land Bridge) and south (e.g. all of the deglaciated area south of British Columbia, Canada) of the ice sheets [35–37]. Other unglaciated areas (e.g. Haida Gwaii in British Columbia) could also have been micro-refugia [38,39]. In this context, distinct demographic scenarios can be tested. Under a first scenario whereby populations expanded north from a single southern refugium, we predict: *i*) a latitudinal decrease in genetic diversity from south to north along with a pattern of isolation-by-distance (IBD), and *ii*) ancestral populations located in areas south of the ice sheets. Under a second scenario, populations expanded south from a single northern refugium, and we predict the opposite geographic pattern. The third scenario corresponds to the survival of populations in different refugia where we predict: *i)* the existence of clearly distinct genetic clusters, and *ii)* postglacial gene flow with signatures of secondary contacts, with contact zones displaying higher genetic diversity through postglacial admixture between different genomic backgrounds.

In order to test these alternative scenarios, we generated genome-wide data from nearly 2,000 Coho salmon from California to Alaska, one of the most extensive genomic datasets for a non-model vertebrate species to date. First, to resolve the species demographic history, we used a modelling approach that accounts both for barriers to gene flow affecting migration locally, and for linked selection affecting the rate of drift. Second, we hypothesized that demographic history shaped the pattern of deleterious mutation load, both within and among populations. We hypothesized that postglacial re-colonisation influenced levels of standing genetic variation and favoured the accumulation of deleterious mutations at the expansion front. In these conditions, we predicted that neutral genetic diversity should decrease as a function of the distance from the ancestral populations.

## Results & discussion

### Latitudinal gradient in nucleotide diversity

**Fig 1:**
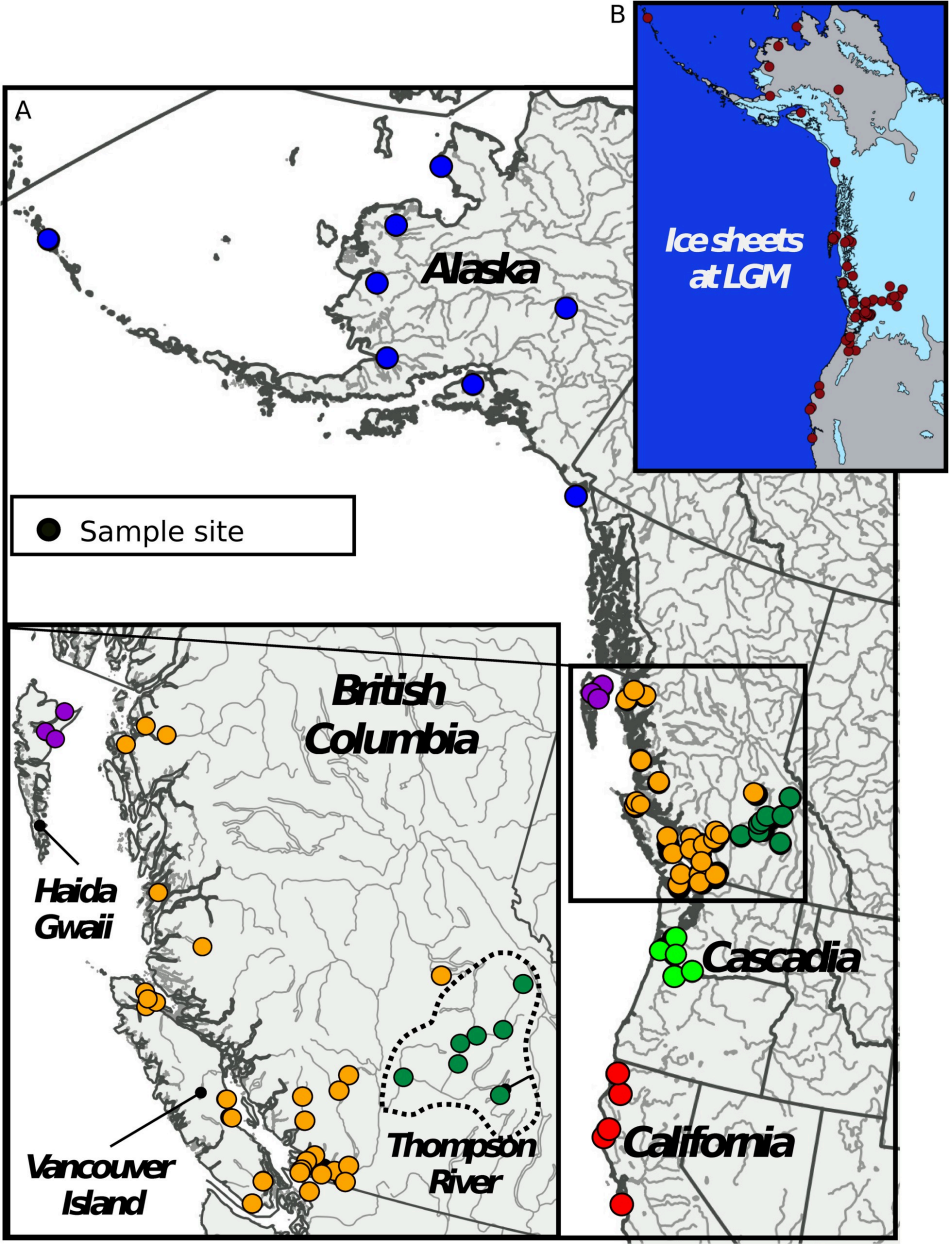
Sampling design. A) Sampling locations of 58 Coho salmon populations distributed across the species’ North American range. Each dot represents a sampling location. B) Map showing the extent of ice-sheet during the Last Glacial Maximum 13 Kya. Map drawn with Qgis software using data from [94, 95].

**Fig 2:**
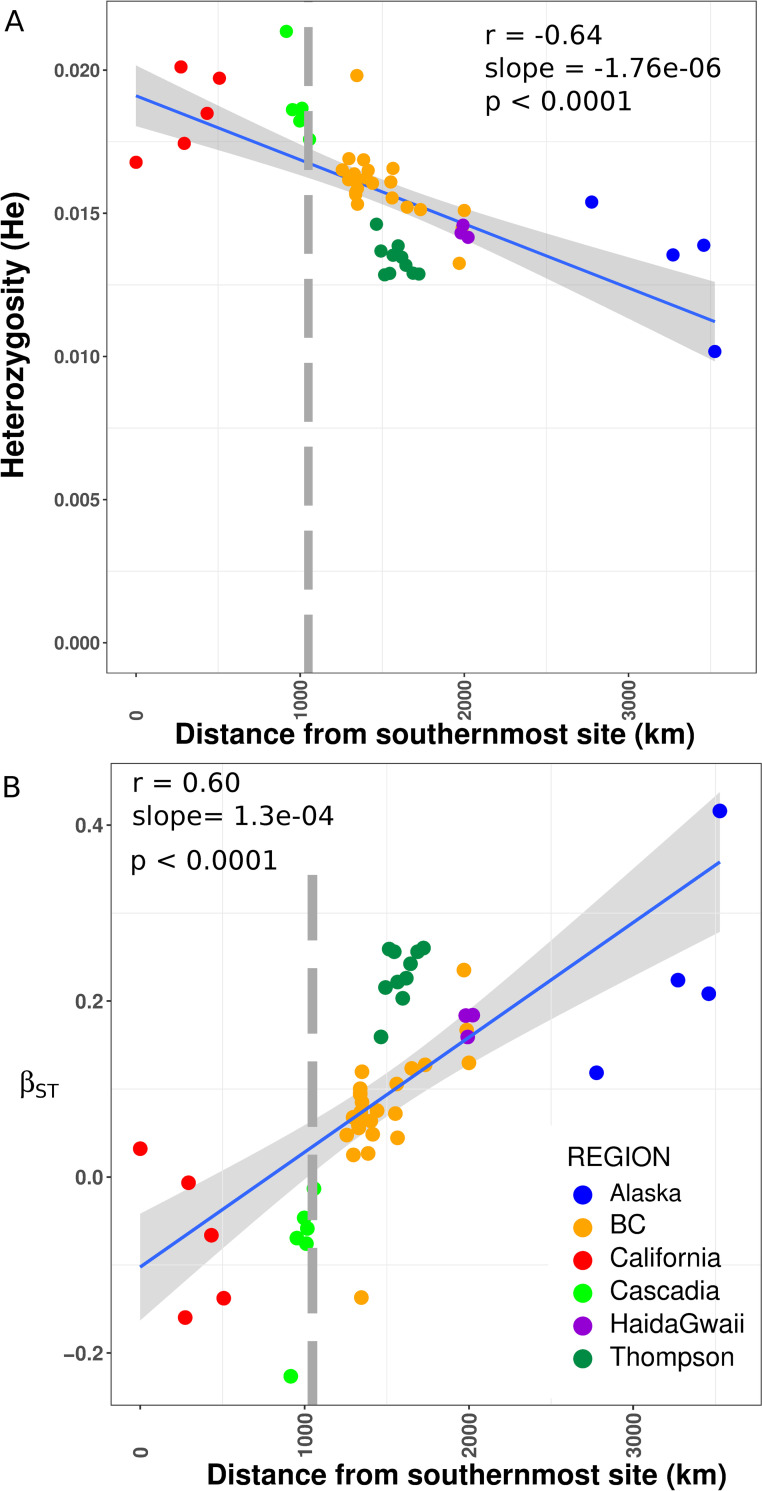
Genetic diversity and differentiation. A) Linear relationship between expected heterozygosity and distance from the southernmost population located in California. B) Linear increase in genetic differentiation as measured by β_ST_ as a function of the distance from the southernmost population located in California. Negative values indicate the most likely ancestral population. The relationship in A and B was tested using linear models. The grey vertical bar in panel A and B indicates the approximate location of the southern limit of the ice-sheet at the end of the last glacial maximum. The coefficient of correlation (r) is indicated for each plot. The grey shaded area along the regression line corresponds to the 95% confidence level interval obtained from the linear models applied to each dataset.

To investigate the distribution of genetic diversity and population differentiation, we sampled 1,957 Coho salmon from 58 sampling locations throughout their entire North American range from California to Alaska (mean n = 34 fish per location, **Fig 1A, S1 Table**). Samples were genotyped using a genotyping-by-sequencing (GBS) method that generated 82,772 high quality filtered single nucleotide polymorphisms (SNPs). Consistent with earlier work based on non-genomic data, our analyses indicate that the southern-most populations are the most ancestral and suggest that a single southern refugium explains most of the observed patterns in the distribution of genetic diversity. Under a single refugial expansion scenario, one would expect genetic diversity to be highest in the ancestral refugium and a linear decrease as a function of the distance from the source, as documented in humans [40]. To test this prediction, we plotted the distribution of genetic diversity as a function of the distance to the southernmost sample in our data. As expected, levels of genetic diversity (observed and expected heterozygosity, π_SNP_) were highest in formerly deglaciated refugial areas in the south (California, Cascadia, **Fig 2A, Fig 1B**) and decreased as a function of distance from the southernmost site up to Alaska (r = 0.64, p < 0.0001**, Fig 2A, S1 Fig, S2 Fig**). Two populations however deviated from this linear pattern (Clackamas R in Cascadia and Upper Pitt R. in BC) and displayed higher genetic diversity than the Southern samples. We hypothesized that these two rivers may have undergone recent admixture from differentiated genetic clusters, locally increasing genetic diversity. Recent admixture between populations from several refugia can produce increased genetic variation [41], which could explain why most ancestral populations (relict populations from the refugia) are not systematically those displaying the highest genetic diversity. The Thompson River watershed (Thompson R. hereafter) in southern British Columbia was an exception to this latitudinal pattern and displayed the lowest average level of regional genetic diversity of all sampling locations, which we hypothesized resulted from bottlenecks (see below). The remaining samples from British Columbia were intermediate in genetic diversity.

### Present-day southern populations are the most ancestral

To help discriminate a single versus two population refugia, we aimed to identify the most ancestral populations among all samples. Under a single scenario refugium, the ancestral populations should be located preferentially in one area (e.g., South or North), whereas under a two-population scenario, we expected ancestral populations to occur in different areas located at both edges of the species’ distribution range (Beringia covering most of Alaska and the Yukon being known as an important glacial refugium for many species (reviewed in [2–3])). To discriminate between these alternative scenarios, we used two simple summary statistics, namely (1) the distribution of singletons among populations, and (2) the βst index recently developed in [42]. It was previously shown that older populations are expected to have accumulated a higher density of singletons [43]. Therefore, counting the number of singletons by sampling site after correcting for sample size (see methods) and averaging by regional groups may help identifying older populations. Alaska and Cascadian samples contained the highest number of singletons, with respective mean values of 753 (sd = 116) and 650 (sd = 152) singletons per site, consistent with the hypothesis of a refugium being present in these regions. Thompson and Californian samples had the fewest number of singletons (n_MEAN_ = 131 and 131 respectively). All regions differed significantly in their distribution of singleton (p < 0.0001) except the comparison between Thompson and California **(S3 Fig**) The observed signal in the Thompson was consistent with our above observations of reduced diversity. Similarly, we hypothesized that such reduced number of singletons in a few Californian samples reflects recent and strong population declines in some of these populations [44,45] (see also S1 Text). The similarity of the singleton distributions for both Cascadia and Alaska further emphasized the need for additional analyses (i.e. modeling approach implemented below) to help disentangle the scenarios of one versus two refugia.

Next, we used the β_ST_ coefficient to identify ancestral populations [42]. Unlike *F*_ST_ estimates [46], β_ST_ can account for the non-independence among populations and negative values are indicative of ancestral populations [42]. More specifically, the β_ST_ coefficient will be negative when a population has accumulated many private alleles at low to intermediate frequencies and the intensity of this value is dependent on the number of sampled populations [42]. As for singletons, older populations and populations of larger effective size have more time to accumulate rare alleles, resulting in negative values, as illustrated in human samples from Africa (see details in 42). Here, the β_ST_ indicated that the likely ancestral populations were in previously unglaciated areas corresponding to Cascadia (n = 5 localities), California (n = 3 localities) as well as one site from southern British Columbia **(Fig 2B, S2 Table**). A linear decrease in β_ST_ with distance from the southernmost site was observed (r = 0.60, slope = 1.03e-04, *p* <0.0001) as expected under isolation-by-distance (IBD). Support for this IBD pattern was also observed using *F*_ST_ (**S4 Fig**, r = 0.66, slope = 4e-05, *p <* 0.0001) as well as Mantel tests (r = 0.64, *p < 0*.*0001*; and r = 0.72, *p* < 0.0001 when removing Thompson R. populations). As for genetic diversity, the river CLA in Cascadia and UPT in British Columbia departed from the pattern and displayed the most negative coefficients (**Fig 2B**). Finally, average pairwise *F*_ST_ across all populations was 0.095 and varied from 0.002 to 0.334 (**S5 Fig**), typical of anadromous species connected by gene flow [47].

**Fig 3:**
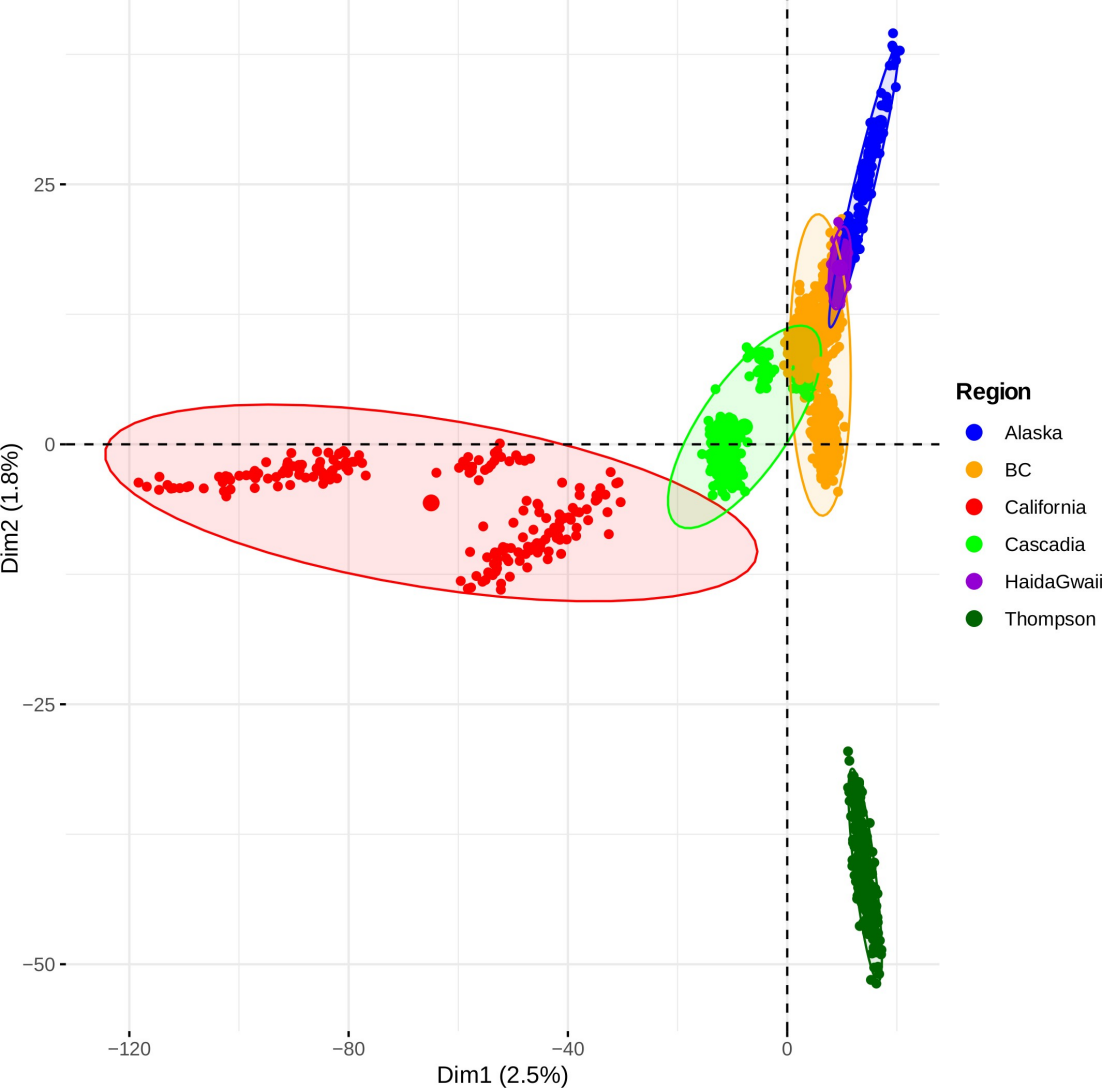
Genetic structure and gene flow. Principal Component Analysis (PCA) summarizing population genetic structure among 1,957 individuals based on the principal component axis 1 and axis 2. Each point represents an individual and the colours represent the major regional groups. The larger point represents the barycentre of each group.

To further confirm the number of putative refugia and better understand the extent of divergence among populations, we performed a principal components analysis (PCA). PCA can be used to summarize the distribution of genetic diversity with different expectations under discrete models of population genetic structure (as expected with different refugia) versus IBD under a one- or two-dimensional habitat model. Assuming K clusters, we expect that the top K-1 PCA axes should discriminate all these clusters [48]. On the contrary, under models of IBD, the top two axes should be correlated with geographical axes such as the longitude and latitude of populations’ locations [49]. Consistent with this latter hypothesis, we observed that the first axis was strongly correlated with latitude (r = 0.77, *p =* 2e-16), whereas the second axis was strongly correlated with longitude (r = -0.54, *p* = 2e-16). The result of the PCA (**Fig 3, see also S6 Fig**) is therefore consistent with a South to North spatial pattern of genetic structure, with the most divergent samples found in California, and a second geographical gradient from East to West, mainly reflecting the pronounced differentiation of the Thompson R. populations. Along these axes, populations followed an IBD pattern. These results were also supported by an MDS analysis (**S7 Fig**). A model-based analysis of population structure implemented in LEA [50] failed to reveal a clear number of distinct populations (K value). Instead, K values ranging from 30 to 60 all fit the data well (**S8 Fig**), providing little insight on the number of ancestral clusters in the data due most likely to the confounding effect of IBD [51]. While all these summary statistics lend support to the hypothesis of an expansion from a single ancestral refugium, a formal testing of alternative scenarios is required as the signature of secondary contact can be transient and both models of secondary contact and stepping stone can converge to the same IBD signal and apparent demographic equilibrium [10].

Finally, using Treemix [52], we found that 99.1% of the total variance in allele frequency among populations can be explained by a single tree (**S9A–S9C Fig**) with four significant migration events **(S9D and S9E Fig**). California populations displayed pronounced genetic drift, corroborating the PCA results. Populations from Alaska (MSL River) and Thompson R. also displayed higher genetic drift, in line with our above results. We note that populations followed the south to north arrangement, with the samples from Cascadia displaying less drift than those located further north.

### Colonisation wave from a single major southern refugium

In order to formally assess the occurrence of one or more refugial origins for contemporary populations, we performed explicit model-based inferences of population divergence scenarios using ∂a∂i [53] and Fastsimcoal [54]. ∂a∂i was used to perform pair-wise population comparisons in order to 1) consider the confounding effects of linked selection and barriers to gene flow, and 2) calibrate a multi-population model to be used in Fastsimcoal. Under a model of a single refugium, either northern or southern, we expect to find support for a model of recent or ongoing divergence with gene flow (isolation with migration or IM) when comparing neighboring pairs. Following this hypothesis, IM models should be the best supported because the divergence time between the different populations should be recent, thus resulting in recent inferred divergence times. In addition, these populations are expected to still be connected by ongoing gene flow. Under a scenario with two ancestral refugia, we predict that comparing Alaskan populations (i.e. assuming that Coho survived in the Beringian refugium) to other genetic groups should reveal signals of secondary contact (SC). In addition to this evidence for postglacial gene flow, one can expect to infer a long divergence time between Alaskan populations and the rest of the populations. Finally we completed our inferences with a null model of strict isolation (SI) where populations would never have exchanged gene and a model of ancient migration (AM) where populations exchanged gene at the early stage of divergence only.

To reduce the number of pairwise comparisons and thus reduce the possibility of spurious results, we pooled individuals into higher order groups based on the PCA results (**Fig 3** and **S10 Fig**). The following six groups were constructed (named according to geographic labels): 1) California group 1, 2) California group 2, 3) Thompson 4) Cascadia 5) British Columbia and 6) Alaska. To avoid over-fitting of complex models, we performed model selection hierarchically. First, we compared all models (AM, IM and SC, SI) represented in **S11 Fig** assuming a constant size after divergence. Second, after choosing the best model based on ΔAIC, we incorporated changes in population size to estimate demographic parameter. All reported demographic parameter estimates are based on this last round of analysis. We compared Alaskan samples to all other groups (n = 4 pairwise comparisons). Then we compared the Thompson R. populations, which display an unusual pattern of reduced diversity and divergence from all other groups (n = 5 pairwise comparisons), resulting in a total of 9 pairwise comparisons.

Our inferences provided considerable support for an expansion from a single major refugium. Indeed, our analyses support models of divergence with ongoing gene flow between Alaska and all the other groups in three out of the four pairwise comparisons involving Alaska (**S12 Fig** for model fit and residuals and **S3 Table**; ΔAIC > 10; with the notable exception of Thompson, see below). These results are inconsistent with a major Beringian refugium, or at least a contribution of these northern populations to the present-day within-species variation. One out of the 4 comparisons (“California 1” versus “Alaska”) provided support for secondary contact. This result, was contrary to our expectations and highlights the well-known difficulty of distinguishing models of secondary divergence from primary divergence. Indeed, these two classes of models can quickly converge to the same state as observed here, namely large-scale isolation by distance and apparent equilibrium [10]. Interestingly, when any group is compared to Thompson R., all inferences find unambiguous support for postglacial secondary contact (**S3 Table**), suggesting that the Thompson R. might have act as a second refugium following the southern expansion and has been isolated from the main distribution. The relative isolation of the Thompson R. would therefore be consistent with the lower genetic diversity observed in each local population within this area. It is noteworthy that in all models with support for secondary contact, the relative time of secondary contact represented on average 10% of the total divergence time. This proportion provided ideal statistical power to discriminate among models of divergence with continuous gene flow versus allopatric divergence followed by gene flow. Indeed, the signal of allopatric divergence is expected to be lost if gene flow was initiated too long in the past [17,55].

### Statistical support for ancestral expansion followed by recent demographic declines across all populations

**Fig 4:**
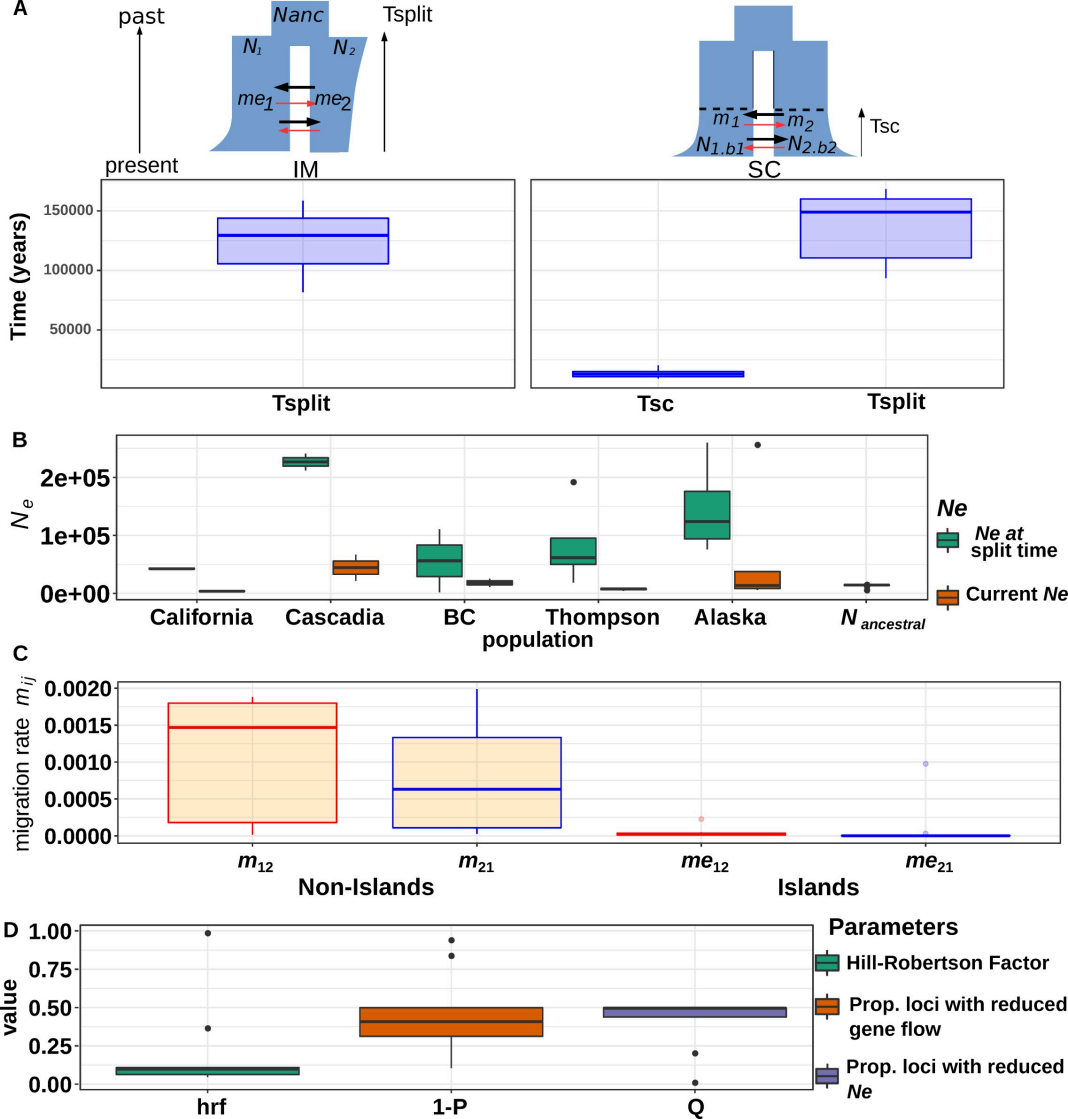
Inferences of demographic history using ∂a∂i. A) Estimates of divergence time (in years) between each major region as inferred by ∂a∂i under the best model (displayed in blue) based on SNP data. Plot displayed the parameter estimates for the best model for all pooled samples (IM = comparison between Alaska and all samples but Thompson SC = comparison between Thompson and all other samples as well as California versus Alaska) B) Estimates of effective population size *Ne* for each major region as inferred by ∂a∂i under each best demographic model. Effective size are based on the best model including population size change. Three extreme values (*Ne* > 2e6) were removed for readability). C) Estimates of migration rate among populations in neutral regions (*m*) of the genome and in areas of restricted recombination (*m*_*e*_) or islands of divergence. D) mean Hrf (Hill-Robertson factor) inferred across all comparisons. The Hrf estimates the extent of Ne reduction in areas affected by linked selection. Values are the averaged from multiple pairwise comparison across regions. Raw data are provided in S4 Table.

We added a second layer of complexity by incorporating changes in population size in the models namely, expansions or bottlenecks. We hypothesized that these changes are likely to arise following postglacial expansion or through serial founder events during the recolonization process. We chose the best models previously identified through weighted AIC (**S3 Table**) and performed a round of parameter estimation. A salient result was that all populations exhibited a signature of expansion from the ancestral refugial population, with the effective population size at the divergence time being approximately 12 times higher than the ancestral population (**Fig 4**). Then all populations declined to approximately 10–12% of their initial size (**Fig 4**). More precisely, assuming a generation time of 3 years [56] and a mutation rate of 8e^-9^ bp/generation [57], our analysis inferred remarkably similar ancestral effective population sizes across all our pairwise comparisons (approximately 15,000 **Fig 4, S4 Table**). Then we recovered a signal of expansion, with stronger expansion signals inferred in Cascadia and Alaska (**Fig 4**). However, we observed some uncertainty in these parameter estimates (**S5 and S6 Tables**). This was expected because of the limited information available from the site frequency spectrum [57–60]. Moreover, we hypothesized that the life history trait of the species, particularly its strong natal homing behaviour [61] may result in different local evolutionary trajectories. Admittedly, such unmodeled process may further complicate the interpretation of demographic parameters.

### Demographic inferences support glacial population isolation and recent recolonization

Our demographic inferences provided highly consistent divergence time estimates among all models and populations (**Fig 4)**, with an estimated divergence time of 140 Kya (average = 107 KyA under IM and 137 Kya under SC; **Fig 4, S5 and S6 Tables**) and suggesting a nearly simultaneous subdivision and colonization of the Northern range, likely before the Wisconsin glaciation. Accordingly, the median time of secondary contact (SC) among all models involving the Thompson R. was 13.5 Kya [min 9,200 –max = 20,100], corresponding to the onset of the last glacial retreat (**Fig 4**), supporting the idea that the Thompson R. has evolved in isolation from the remaining populations for a long period of time.

Our models account for the confounding effects of selection at linked sites and that of the accumulation of local barriers to gene flow in the genome, two potential confounding factors that can lead to erroneous interpretations when they are not explicitly taken into account [12,17]. All models incorporating linked selection and restricted introgression along the genome always received the highest support. Here we modeled the effect of linked selection assuming that it can be approximated as a reduction of *Ne* in regions of low recombination [15]. Our inferences revealed that on average, 40% of the genome was undergoing reduced effective population size (Hill Robertson Factor in **S4 Table, Fig 4**). Accordingly, the *Ne* values were reduced to approximately 20% of their initial values depending on the considered population (i.e. Hrf factor in **Fig 4**). Obviously, the magnitude of the reduction would require further investigation as quantifying the effect of linked selection is likely more complicated than a simple rescaling of *Ne*. Still, these results provided increased support for a role of linked selection affecting the estimation of demographic parameters [17,62] and potentially shaping the species’ genomic landscape. Quantifying how background selection affects the species’ genome and shapes the genomic landscape of divergence is beyond the scope of this study and awaits further investigation. Finally, intrinsic barriers to gene flow reduced the estimated migration rate to nearly zero inside genomic islands and affected approximately 47% of the genome (**Fig 4, S6 Table**).

To consolidate inferences made from ∂a∂i we constructed a global model based on ∂a∂i parameter estimates in Fastsimcoal v2.6. To limit the confounding of barriers to gene flow [11], linked selection [12] or gBGC [16], we restricted our jSFS to areas of high recombination, as inferred by LDHAT [63] from whole genome sequences [64]. Results largely validate our ∂a∂i inferences and provide rich information confirming strong population size reduction in California, Thompson, Alaska while revealing expansions in BC and Cascadia along with recent divergence of all these populations (details in **S1 Text, S7 Table)**.

In summary, our results best supported a scenario whereby contemporary populations mainly originated from a single major ancestral refugium located south of the ice sheets in Cascadia/California, followed by a postglacial expansion and population divergence along the South to North recolonization axis. As a consequence, we expect that the South-North expansion should favour the accumulation of deleterious mutations at the expansion front toward the north [65,66], but also with the possibility for a pulse of deleterious variants from the isolated populations from the Thompson River watershed when they came into secondary contact with other expanding populations.

### The recent evolution of Ne shaped the current mutation load

**Fig 5:**
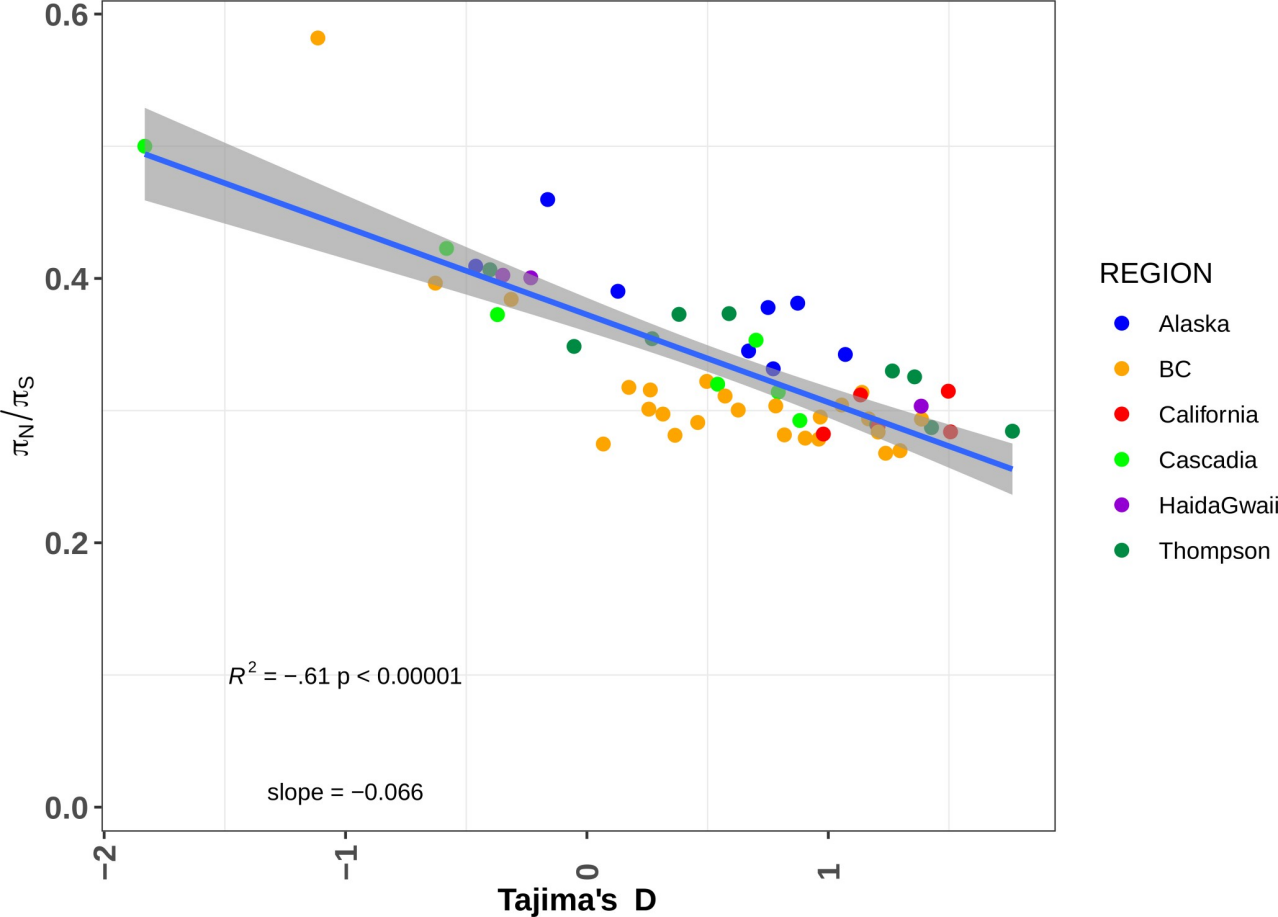
Recent demographic history shaped the deleterious mutation load: Correlation between Tajima’s D and the deleterious load (π_N_/ π_S_). R^2^ = coefficient of determination of the linear model.

Our above demographic reconstruction revealed expansion from a major southern refugium. Consequently, we hypothesized that such a history has generated significant differences in the deleterious mutation load among populations, consistent with patterns documented in human populations [65–67]. Therefore, we hypothesized that populations having undergone a strong expansion following a bottleneck should display a higher load than populations of more constant size. Under this hypothesis we predicted a negative correlation between Tajima’s D, a classic population genetics statistic capturing population demography changes, and the ratio of non-synonymous to synonymous diversity (π_N_/π_S,_ **S8 Table**), a commonly used proxy for the deleterious mutation load [68]. Accordingly, π_N_/π_S_ was significantly and negatively correlated with Tajima’s D (**Fig 5,** R^2^ = 0.65, *p = 1e-15*, slope = -3.4). Considering our demographic inferences, this result illustrates how the postglacial recolonization changed local effective populations and shaped the present-day deleterious load variation among these populations. Interestingly, even after excluding the two most highly loaded populations (Clakamas and Upper Pitt River in Cascadia and BC respectively), the relationship between the π_N_/π_S_ and the Tajima’s D still remain strong (R^2^ = 0.42; *p* = 4e-8, slope = -2.7). Our results suggest that the Clackamas River populations exhibit high levels of admixture with the Thompson R populations (details in **S13 Fig**). As a consequence, we hypothesize that the post-glacial secondary contact with this population contributed to the introgression pulses of maladaptive alleles that are still detectable nowadays. This raises exciting future research questions regarding the role of past introgression and the maintenance of deleterious alleles [69]. It must however be noted that the Tajima’s D values were more widely distributed than expected under a strict model of population expansion from the South (assuming a linear decrease with distance, **S14 Fig**), suggesting additional sources of variation that remain to be identified and their effect evaluated.

### Genetic surfing of deleterious mutations during the recolonization process

One possible consequence of postglacial recolonization is that neutral and deleterious mutations may increase in frequency (i.e.surf) at the expansion front [65]. This situation occurs because populations at the expansion front exhibit smaller sizes that prevent the efficient purging of deleterious variants. Theory predicts (i) a higher recessive load (measured as the proportion of homozygous derived deleterious mutations) at the expansion front and (ii) an approximately constant total load (which can be approximated by the total number of derived deleterious mutations under an additive model [28,65–67]). We found support for these predictions.

**Fig 6:**
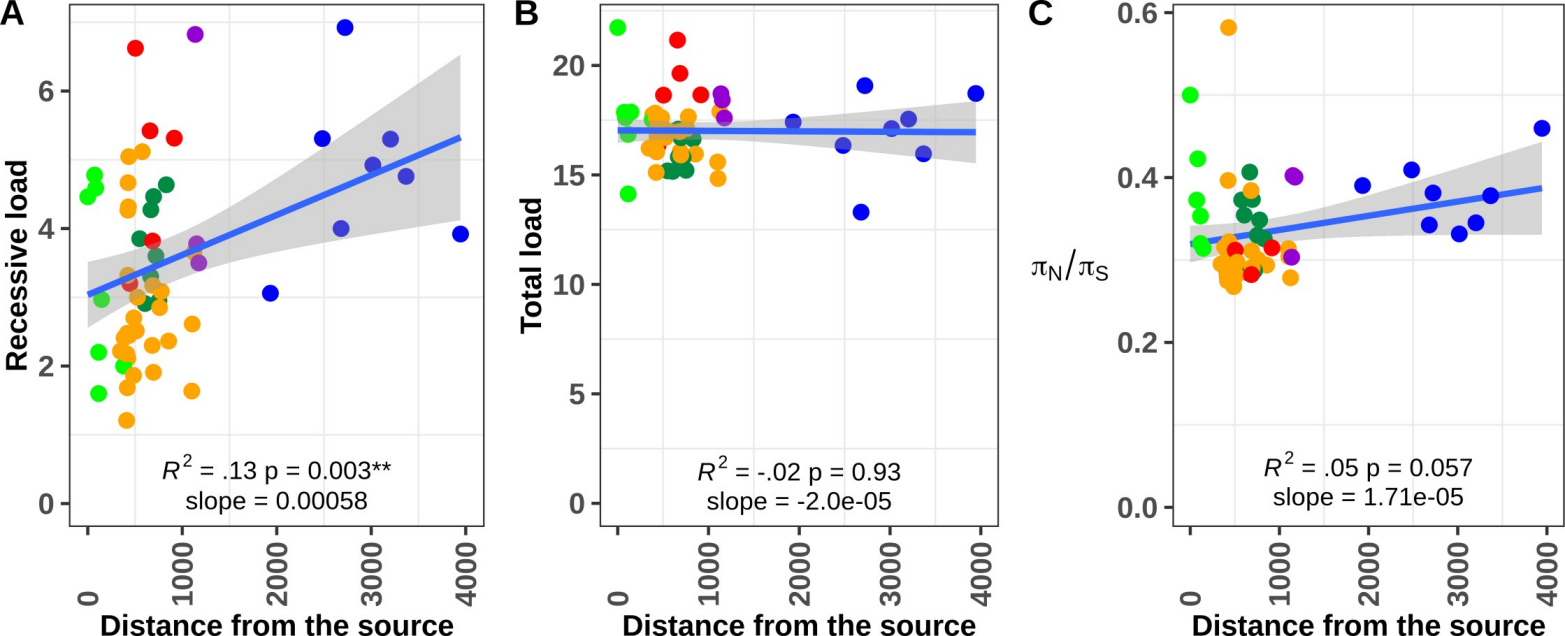
**Expansion history and geographic pattern of deleterious mutation load:** Correlation between the distance to the southernmost site and the deleterious load measured as (A) the distribution of homozygous derived putatively deleterious mutations (i.e. recessive load). B) the total load expected to be approximately constant among all populations) and C) the π_N_/π_S_ ratio. R^2^ = coefficient of determination of the linear model.

First, we found a nearly linear relationship between the distance to the candidate source (defined as the one with the lowest β_ST_ and highest diversity) and the recessive load (linear models, *p*<0.003; R2 = 0.13, **Fig 6A**), validating theoretical expectations from range expansion [67]. This latter analysis was performed with PROVEAN [70] based on a total of 1,297 deleterious mutations for which we were able to identify the derived allele using the genome sequences of the 1) Chinook salmon (*Oncorhynchus tshawytscha*) [71], 2) Rainbow trout (*O*. *mykiss*) and 3) Atlantic salmon (*Salmo salar*) [72] (see methods). Second, and as expected, the total load was not significantly correlated with the distance to the source (R^2^ = 0.02, *p* = 0.96, **Fig 6B**). Since PROVEAN is primarily designed for Human and Mouse (although it has been used successfully in non-mammalian studies [73,74]), we also plotted the distribution of π_N_/π_S_ as a function of the distance to the southernmost site. The π_N_/π_S_ ratio was correlated with the distance from the southernmost site **(Fig 6C**, p = 0.057; R^2^ = 0.05, for all populations). After excluding Clackamas and Upper Pitt populations (hypothesized to display increased load due to admixture), the correlation significantly increased (R^2^ = 0.2, *p* = 0.0003, slope = 2.395e-05*)*. Third, we observed significant differences in the derived allele frequency (DAF) at predicted deleterious non-synonymous mutations among regions (Kruskal-Wallis chi-squared = 100.57, df = 5, *p* < 2.2e-16) as well as among populations (Kruskal-Wallis chi-squared = 638.59, df = 57, *p* < 2.2e-16,) with higher values indicative of less efficient purging (See S15 **Fig to S18 Fig and S9 and S10 Tables for details**). To the best of our knowledge it is the first time that such correlations between different proxies of the load and distance to the putative refugial origin are reported other than in studies on humans [28,66] and a few in plants [75,76] or bacteria [77]. As such, our two major results regarding Tajima’s D and the evolution of expansion load as a function of the distance aligned perfectly with the inferred expansion history and recent change in effective population size in each local population.

Finally, a general prediction from population genetic theory is that deleterious mutations in a heterozygous state should be more frequent than in a homozygous state, especially in populations with higher effective sizes, where selection should be more effective at purging mutations with increased homozygosity [19]. Accordingly, we found that 77% of deleterious mutations were maintained in heterozygous states across all samples, as reported in other species [73]. Also, salmon from Alaska, Haida Gwaii, and California populations harboured a significantly higher number of deleterious mutations in a homozygous state when compared to Cascadia or British Columbia (Wilcoxon-test, *p* < 0.01) **(S17A Fig, S11 Table**), a result that is in line with our finding of increased expansion load in **Fig 6A**. When considering the total load of derived deleterious mutations, we found that, on average, there were indeed significantly more putatively deleterious variants per individual in California, Cascadia, and Haida Gwaii populations than in fish from Alaska, British Columbia, or the Thompson R. watershed (**S10 and S11 Tables, S17B Fig**, Wilcoxon-test, *p* < 0.01), although these differences were modest. Accordingly, we observed that π_N_/π_S_ varies as a function of the levels of nucleotide diversity (π_S_). We indeed found a positive relationship between π_N_/π_S_ and π_S_ (slope = 175.5; R^2^ = 0.45, *p* < 0.0001, **S19 Fig**). We note again the same two populations (Clackamas River & Upper Pitt) were strongly contributing to the linear regression. However, the relationship remained significant after their exclusion (R^2^ = 0.10, *p* < 0.0078). This result provides increased support for our above findings that populations with higher effective population size also displayed a higher deleterious load when considering mildly deleterious variants in heterozygous states (those that are more likely to contribute to π_N_). This suggests strongly that deleterious mutations are efficiently purged in populations of large *Ne* while mildly deleterious mutations would still segregate at appreciable frequency [23], as observed elsewhere [21,22,26]

The role of historical demography in generating differences in the mutation load at the population level remains debated among evolutionary biologists, particularly among human geneticists [7,9,28,29,78]. Our results are however consistent with the nearly neutral theory and the accumulation of deleterious mutations in populations on the expansion front, thus producing a so-called expansion load [65–67]. It is noteworthy how well our findings mirror the ‘out-of-Africa’ expansion model of human populations [40], despite the fact that the investigated geographic distance for the Coho salmon is an order of magnitude smaller than that involved in the human studies, and that the presence of additional smaller refugia such as Thompson R. (as opposed to the sole African ancestral origin for humans) led to more challenging inferences.

## Conclusion

**Fig 7:**
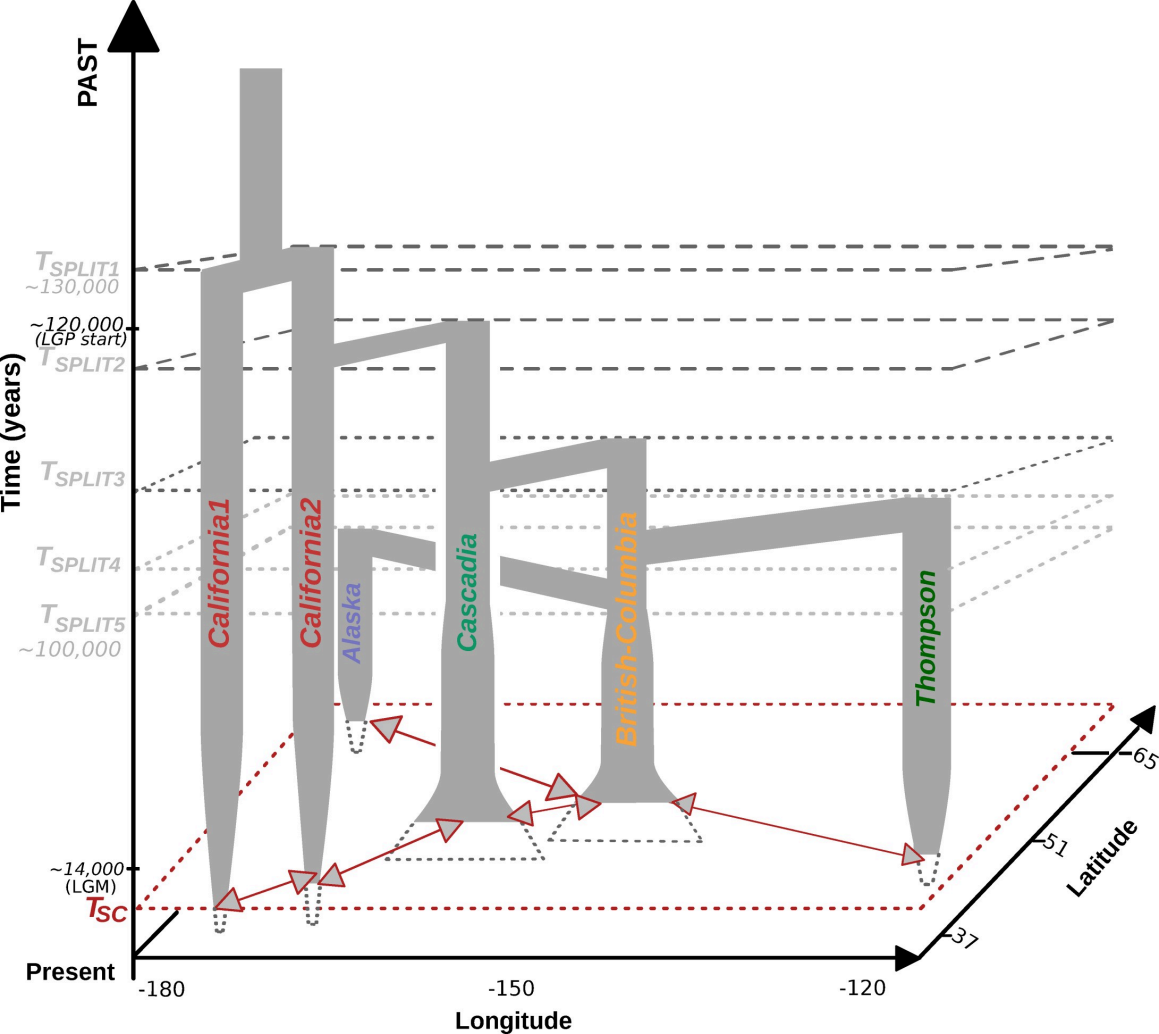
Simplified spatio temporal representation of the coho salmon demographic history based on ∂a∂i and Fastsimcoal results. Each bar represents a population branch. the width of the branch is not proportional to the population effective size and do not account for historical population size changes. The split time are not to scale and geographic positions are approximate. Tsc = Time of secondary contact. Red arrows represents major gene flow events between neighboring populations. Details about gene flow event can be found in S5 Table and S7 Table. LGP = approximate start time of the Last Glacial Period [96]. LGM = Last Glacial Maxima Beringia was unglaciated during most of the pleistocene [97]. See results for details of the grouping.

In this study, we provided rare evidence of how demographic modelling can provide valuable information for conservation genomics. We found considerable statistical support for a recent postglacial demographic history in the Coho salmon, consistent with a main southern refugium, which generated relatively simple latitudinal gradients in nucleotide diversity and mutation load. Complex demographic processes including past population expansions or periods of isolation and secondary contact, have also been reconstructed with state-of-the-art demographic modelling methods. This was made possible by jointly considering the confounding impact of linked selection and barrier to gene flow on such demographic reconstructions. Altogether, this postglacial history, as well as some additional demographic changes, have influenced the efficacy of selection and favoured the accumulation of deleterious mutations in the most distant populations from the main refugium (**Fig 7**). Using the Coho salmon as a case study, we demonstrated how these approaches are meaningful for conservation genomics and provide opportunities to disentangle the impacts of historical *vs*. contemporary drivers of population declines. Such investigations also allow assessing the distribution of all selected variants, including not only the beneficial, but also deleterious ones.

## Methods

### Genotyping by sequencing

A total of 2,088 individuals were collected from 58 sample sites located along the Pacific coast from California to Alaska (S1 Table and **Fig 1**). DNA was extracted from all individuals and sequenced using a GBS method (protocol detailed in [79]). Reads were aligned to the Coho salmon reference genome v1 (GCF_002021745.1) using bwa-mem 0.7.13 [80]. Samtools v1.7 [81] was used to keep reads with a mapping quality above 20, remove supplementary alignment and unmapped read. Variants were then called with Stacks v1.46 [82]. To do so, the module “pstacks” was used with a minimum depth of 5, and up to three mismatches were allowed in catalog assembly. The module “populations” was run to produce a vcf file that was filtered with a custom python script. We performed stringent filtering to remove SNPs that were 1) genotyped in less than 60% of the individuals; 2) at a mean depth of sequencing (averaged across all individuals) below 7, and 3) with observed heterozygosity above 0.60, thus resulting in 93,000 SNPs. The pipeline for SNP calling is available on github at https://github.com/enormandeau/stacks_workflow/releases/tag/coho_demography_paper. Next, we removed any individuals with more than 5% missing data and finally only kept SNPs present in at least 95% of the individuals yielding a total of 82,772 filtered SNPs for 1,957 individuals. Remaining filtration was done according to the requirement of each analysis performed below. To perform explicit demographic analyses based on the site frequency spectrum, the likelihood approach implemented in ANGSD v0.930 [83] was used instead of the commonly used Stacks pipeline because it is less biased than the genotype calling approach [84]. We first used the SAMTools model [81] in ANGSD to estimate genotype likelihoods from BAM files using only reads with a minimal base quality score of 20 and a minimal mapping quality score of 30 across all individuals. We further filtered out reads with a depth below 5 and above 100. Given the high residual tetrasomy in the species, the upper bounds enabled removing the high rates of paralogs that would otherwise generate an excess of high frequency shared variants in the jSFS. We verified that such variants were effectively removed in all jSFS before ∂a∂i fitting. We further constructed 1D SFS for all localities to then compute Tajima’s D value as a summary of recent effective population size change.

### Genetic diversity and ancestral populations

For each sampling location we estimated the observed heterozygosity and π using vcftools 0.1.16 [85] and Hierfstat [86]. The most likely ancestral populations were identified using β_ST_ [42]. A total of 1,000 bootstraps was performed to obtain the 95% confidence intervals around the β_ST_. Weir and Cockerham’s *F*_ST_ estimator θ [46] was computed in vcftools. We measured the relationship between observed heterozygosity, β_ST_, *F*_ST_ and the distance to the southernmost site using linear models with the lm() function implemented in R. We also verified the relationship between *F*_ST_ and the distance to the southernmost site using Mantel tests with 10,000 permutations. Vcftools was also used to identify singletons (i.e. variants present in one single individual across the whole dataset). The power to discover singletons is however dependent upon the sample size. Therefore, we reduced the size of each population to the smallest size of 13 by randomly sampling individuals in each population. We repeated the procedure 200 times to obtain standard deviations of the distribution of singletons (S3 Fig). We then computed the averaged number of singletons at the regional level as well as levels of private and shared polymorphism (S2 Fig and S3 Fig). We tested the significance of differences in singletons distribution across the 200 replicated using a pairwise Wilcoxon test corrected with the Benjamini Hochberg method for multiple tests [87].The scripts for ANGSD and differentiation analyses are available on GitHub at https://github.com/QuentinRougemont/utility_scripts

### Population structure, admixture and gene flow

Levels of ancestry and admixture proportions were inferred with the snmf function implemented in the R package LEA [50], allowing only less than 5% of missing data. We then kept a set of SNPs in approximate linkage equilibrium by removing pairs of variants with r^2^ greater than 0.2 (option—indep-pairwise 50 10 0.2) resulting in 40,632 SNPs. K-values ranging between 1 and 60 were tested and cross-validations were obtained using the cross-entropy criterion with 5% of masked genotypes. The default value for the regularization parameter was used to avoid forcing individuals into groups and hence underestimating admixture. Similar results were obtained from Admixture [88] and are not presented here. The genetic relationship among all salmon was assessed using a PCA with the R package ade4 [89] based on the LD-pruned dataset (40,632 SNPs). We used a 1% minor allele frequency (MAF) threshold and allowed less than 4% missing data. Formal tests of admixture were performed using Treemix [52] using the LD-pruned dataset of 40,632 SNPs and without any MAF threshold. An MDS was also constructed using plink and plotted with the ggplot2 [90] R package. We ran Treemix allowing up to 20 migration events and performed 500 bootstrap replicates of the best model to obtain robustness of the nodes. The “best” model was inferred after inspecting the relevant migration edges by measuring the percentage of variance explained as migration edges were added to the tree, as well as by assessing the p-value associated to each migration edge. A total 500 bootstrap replicate runs were performed under the “best” model and under a model without migration to infer the robustness of the nodes. The scripts are available on GitHub at https://github.com/QuentinRougemont/treemix_workflow

### Explicit demographic inferences

We tested alternative hypotheses of divergence from a single southern or northern refugium versus divergence from two or more refugia (e.g. Alaska & California). In the first case, by comparing different regional groups, we expected a model of divergence with ongoing migration (IM) to be supported, or alternatively a model of divergence with ancient migration if gene-flow has stopped recently (AM) following the divergence and recent colonization of rivers. In the case of divergence into multiple refugia we expected models of secondary contact between putative groups to be best supported. We further expected postglacial gene flow between the historical refugia to leave detectable traces that should correspond to the signals of secondary contact. In the unlikely case of no gene flow between regional groups, we expected a model of strict isolation to be most likely. All divergence scenarios are represented in S12 Fig and initially described in [17,91]. The four major models tested included a model of Secondary Contact (SC), a model of Strict Isolation (SI), a model of Ancient Migration (AM) and a model of Isolation with Migration (IM).

The models shared the following parameters: the ancestral populations of size N*anc*, splits at time *T*_*split*_ into two daughter populations of size *N*_1_ and *N*_2_. Under the SI model, no gene flow occurs between the two populations. Under AM, gene flow occurred between *T*_*split*_ and *T*_*am*_ and is followed by a period of strict isolation. Under IM, gene flow occurs at a constant rate at each generation between the two populations. Gene flow can be asymmetric, so that two independent migration rates *m*_*12*_ (from population 2 to 1) and *m*_*21*_ (from population 1 to 2) were modeled. Under the SC model, the population evolved in strict isolation between *T*_*split*_ and until *T*_*sc*_ where a secondary contact occurs continuously up to present time. Gene flow is modeled as *M = 2Nref*.*m*. In ∂a∂i, heterogeneous introgression was modeled using two categories of loci occurring in proportions *P* (i.e., loci with a migration rates *M*_*12*_ and *M*_*21*_) and *1-P* (i.e., loci with a reduced effective migration rates *Me*_*12*_ and *Me*_*21*_) across the genome. The same procedure was used to account for linked selection by defining two categories of loci with varying effective population sizes (proportion 1-Q of loci with a “neutral *N*_*e*_” and a proportion Q of loci with a reduced effective population size due to either selection at linked site). To quantify how linked selection affects reduced *Ne*, we used a Hill-Robertson scaling factor (Hrf) to relate the effective population size of loci influenced by selection (*Nr = Hrf* * *Ne*) to that of neutral loci (*N*_*e*_). A hierarchical approach was used to avoid over-fitting: first we compared models assuming constant effective population size. Second, the best identified models were modified to incorporate population expansion or decline, as expected given the observed distribution of genetic diversity. Population expansion was implemented using two additional parameters for population 1 and population 2, allowing each population to either grow or decline exponentially.

Models were fitted using the diffusion theory implemented in ∂a∂i [53] and includes the effect of linked selection and barriers to gene flow as detailed in [17,91]. ∂a∂i uses the SFS as a summary of the data. For a given demographic model, the SFS is computed using diffusion approximation and compared to the empirical SFS using AIC. Here, we started from the whole file containing 200,000 SNPs and used one single SNP per GBS locus and filtered the data to minimize missing data. No MAF filter was used and singletons were kept to avoid ascertainment bias in estimates of demographic parameters. For each model, 20 independent replicate runs were performed and only models with the lowest AIC and ΔAIC were kept. A model was classified as “ambiguous” and not used for parameter estimation if ΔAIC between the best model and second-best model was below 10. The Godambe information criteria was initially used to evaluate parameter uncertainties based on 100 bootstrapped, which resulted in large confidence intervals in a few pairwise comparisons. Therefore, we then performed parametric bootstrap. More specifically, we converted ∂a∂i estimates into ms [92] estimates to construct a set of 100 datasets over the different categories of the SFS (i.e. neutral SFS, SFS with reduced rate of migration, SFS in low recombination areas undergoing linked selection, SFS in areas of normal recombination) that were summed and then fitted with ∂a∂i. The new estimates were then converted into demographic units to obtain the 95% confidence intervals. The whole pipeline is available at https://github.com/QuentinRougemont/DemographicInference.

### Inferring a global divergence history

While the ∂a∂i analyses provided valuable results regarding the demographic history and how it is confounded by linked selection and barriers to gene flow, this analytical framework is currently limited to pairwise comparisons, which does not allow painting a global scenario of the species divergence history. Therefore, we build upon the ∂a∂i models and other observed statistics to construct a global scenario of population expansions from the South to the North with a postglacial secondary contact between Thompson and the main distribution. This scenario was then tested using Fastsimcoal v2.6 [54] and is described in S1 Text.

### Genetic load estimation from the GBS data set

#### Estimating ancestral and derived alleles

We identified derived and ancestral allele using the genomes of three outgroup species, the chinook salmon, the rainbow trout, and the Atlantic salmon, to classify SNPs as ancestral or derived. These informations were subsequently used to analyze the load of deleterious mutation. Whole genome data for the chinook salmon (n = 3 individuals) were provided by one of us (B. Koop), whereas rainbow trout (n = 5) and Atlantic salmon (n = 5) data were downloaded from NCBI Sequence Read Archive (rainbow trout, SRA, Bioproject: SRP117091; *Salmo salar* SRA Bioproject: SRP059652). Every individual was aligned against the Coho salmon V1 reference genome (GCF_002021745.1) using GATK UnifiedGenotyper and calling every SNP using the EMIT_ALL_SITES modes. We then used a python script to determined the ancestral state of the GBS SNPs if 1) the SNP was homozygous in at least two of the three outgroups, and 2) match one of the two alleles identified in Coho salmon. Otherwise, the site was inferred as missing and not used in subsequent analyses.

#### Measuring the deleterious load through piN/piS ratio

The ratio of non-synonymous diversity to synonymous diversity is a commonly used metric to quantify the deleterious mutation load e.g. [68]. To do so, we first generated aligned fasta sequences and then computed summary statistics. More specifically, for every individual, we ran GATK v4.1.2.0 on the bam file generated above following GATK best practices. We generated individual gVCF files then combined individuals into populations using the CombinedGVCFs module from GATK and finally performed a joint genotyping using the all-site options. We then reconstructed fasta sequences from VCF files for every individual by reconstructing two genomic sequences with a pipeline modified from [93]. We then used cutSeqGff.py to subsample and clean sequences considering several genomic features (CDS, introns, intergenic regions) from gff files. The resulting alignment was then used to compute the π_N_/π_S_ ratio in sequences containing 4-fold degenerate codons only. We then tested a number of correlations between the load (π_N_/π_S_) and 1) π_S_ used as a proxy of the long-term effective population size; 2) Tajima’s D used as a proxy for historical change in population size and 3) distance to the Southernmost site. Significance of the correlation were tested using the lm() function in R. Plots were drawn using ggplot2 [90]. All pipelines to run GATK and compute deleterious load are available at: *https*:*//github*.*com/QuentinRougemont/gatk_haplotype* *and* *https*:*//github*.*com/QuentinRougemont/piNpiS*.

#### Measuring damaging impact of non-synonymous alleles

While the computation above provides insights into the deleterious mutation load, it does not allow distinguishing among recessive and additive load nor testing for differences in allelic frequencies. We therefore further tested differences in mutation load among populations as follows. The software Provean [69] was used to predict whether a given non-synonymous mutation was deleterious with a threshold score of -2.5 or less using the pipeline available at https://github.com/QuentinRougemont/gbs_synonymy_with_genome. We analysed the data in two ways: first we counted the total number of putative homozygous deleterious alleles per individual as well as the total number of deleterious alleles (both in homozygous and heterozygous states) using: N_total_ = 2 Χ N_homo_ + N_hetero_ [34]. These two metrics are expected to be proportional to the recessive and deleterious load respectively [28,67]. These individual values were then averaged per population and major regional group (i.e., California, Cascadia, British Columbia, Haida Gwaii, Thompson, and Alaska). We then computed derived allele frequencies (DAF) in all sampling locations and across mutation categories (synonymous, non-synonymous, and non-synonymous deleterious) and tested for significant allele frequency differences among populations in non-synonymous and non-synonymous deleterious mutations using Wilcoxon rank sum tests. DAF spectra were constructed for all populations separately. For the ease of visualization, we also constructed DAF spectra by region for a sample of size n = 100 individuals. This size was chosen according to the smallest sample size of the three combined Haida Gwaii populations.

## Ethic statement

A permit number SIRUL 111722 was obtained to work on DNA sequences. (i) No specific guidelines were followed as we did not work with animals per se and obtained all of our samples from a tissue/DNA repository of the department of Fisheries and oceans Canada; (ii) the study was approved by the following committee "Comité de protection des animaux de l'Université Laval (CPAUL)" (iii) the approval number "SIRUL 111722" was the project label at University Laval. Following answering the CPAUL questionnaire, we did not get a permit because we did not need one since there were no animals manipulations involved.

Raw data will be deposit on NCBI together with Short Read Archive (SRA) accession number PRJNA647050. The vcf file is available at dryad doi:10.5061/dryad.h44j0zph8. All code to reproduced the analyses is available at: https://github.com/QuentinRougemont/

## Supporting information

S1 Fig Linear decrease in genetic diversity when considering πSNP as a function of the distance to the southernmost sample site. Each points represents a sample site and is colored by region.
(TIF)

S2 Fig Network of shared and private polymorphism. The branch (grey) represent shared polymorphism between sample site and are proportional to levels of sharing. Each point represents the number of private polymorphism and is colored by region. Computation were based on a sample of size 100 in each region to enable comparison. Regional groups were chosen based on the literature regarding expected ancestral refugia.
(TIF)

S3 Fig Violin plot of singleton distribution averaged over each region after correcting for differences in sample size. Shown is the distribution observed across 200 dataset obtained by randomly sampling individuals across populations. Black dots with errors bars represent the mean ±1 standard deviation.
(TIF)

S4 Fig Patterns of Isolation By Distance. Increasing *F*_ST_ as a function of the distance to the southernmost site. Each point represents a sample site and is coloured by region. The *F*_ST_ was computed between the southernmost site and all other remaining sites.
(TIF)

S5 Fig Summaries of *F*_ST_ values. A. *F*_ST_-based Hierarchical tree depicting relationship among samples. Colors represent the major region. B. Heatmap of *F*_ST_ values among samples ordered from North to South on the X and Y-axis.
(TIF)

S6 Fig Principal Component Analysis recapitulating the relationship among individuals. The Axis 3 and axis 4 are displayed.
(TIF)

S7 Fig Multidimensional Scaling (MDS) plot depicting relationship among individuals. Each point represents an individual site and is colored by region.
(TIF)

S8 Fig Structure and Admixture inferences. A. Admixture Barplot obtained from LEA for various K-values. B. Progressive decrease of LEA cross-entropy criterion. Lower cross-entropy values indicates the number of cluster compatible with the data (here from 30 to 60).
(TIF)

S9 Fig Treemix results. A. Proportion of variance explained (y-axis) as a function of the number of migration edge (x-axis) B. Treemix tree inferred without gene flow. C. Treemix tree inferred with four migration edges. D) Residuals for Treemix tree without gene flow E) Residuals for Treemix tree with four migration edges.
(TIF)

S10 Fig PCA representation of the major groups used in the demographic inferences. The site indicated as “blacklisted” are sites that were not included in the ∂a∂i analyses. These corresponds to potentially admixed sites between BC and Alaska and display a reduced number of individuals to constitute a coherent unit for demographic comparison.
(TIF)

S11 Fig Compared demographic models. Strict Isolation (SI), Isolation with constant Migration (IM), Ancient Migration (AM) and Secondary Contact (SC). The models shared the following parameters: Tsplit: number of generation of divergence (backwards in time). *Nanc*, *N*_*1*_, *N*_*2*_: effective population size of the ancestral population, of the first and second daughter population. *M*_*1*_ and *M*_*2*_ represent the effective migration rates per generation that is (M = 2.Nref.m) with *m* the proportion of population made of migrants from the other population and Nref the size of the reference population. T*sc* is the number of generations since gene flow started (secondary contact) after a period of isolation. T*am* is the number of generations since the two populations have diverged without gene flow until present. Each model is declined in alternative version allowing homogeneous or heterogeneous effective size and homogeneous or heterogeneous gene flow to account for the effect of linked selection (affecting *Ne)* and barrier to gene flow (affecting *m*) respective.
(TIF)

S12 Fig Models and residuals for each best model. Each plot displays the observed jSFS (data), the modeled jSFS (model) and the residuals. Left part: Best model without population size change. Each best model was inferred using ΔAIC and AIC weights. Right panel: The same model as the left but including the possibility for population size change of the diverging daughter populations.
(TIF)

S13 Fig Summary of admixture coefficient. For easier interpretations of admixture coefficient among the 58 samples site a PCA was performed to summarized the distribution of admixture. Populations with lower cos2 contributed weakly to the plot and hence have higher admixture.
(TIF)

S14 Fig Relationship between Tajima’s D and distance to the southernmost site. No significant relationship was observed (p = 0.5, r = -0.07), suggesting that each local population has undergone different evolutionary trajectory in post-glacial time.
(TIF)

S15 Fig DAF spectrum of synonymous, non-synonymous and putatively deleterious mutation aggregated at the regional level for all samples. Data are normalized for a sample of size n = 102 corresponding to the smallest size for the combined samples in Haida Gwaii.
(TIF)

S16 Fig DAF spectrum of synonymous, non-synonymous and putatively deleterious mutation in each locality for all samples. (TIF)

S17 Fig: A) Distribution of the count of homozygous derived deleterious alleles in each major group. B) Distribution of the count of total derived deleterious alleles in each major group (TIF)

S18 Fig Mean derived allele frequencies distribution of deleterious mutation. Displayed are the mean derived allele frequencies of polymorphic deleterious sites in each region +/- 2 standard deviation.
(TIF)

S19 Fig Correlation between π_N_/ π_S_ and π_S_ used as a proxy for long term effective population size of each local population. (TIF)

S1 Table Population name. Abbreviation, with corresponding river of sampling, Region and coordinates (Longitude and Latitude) of each sites used in the GBS data with the number of individuals provided (nb. Inds).
(TIF)

S2 Table β_ST_ values along with 95% confidence intervals for each river from the GBS data (82 K SNPs). 95% confidence intervals obtained after 1000 bootstraps.
(TIF)

S3 Table Model choice results for ∂a∂i. AIC, ΔAIC and AIC weights are provided for each pairwise comparison and model. AM = Ancient Migration, IM = Isolation with Migration, SI = Strict Isolation, SC = Secondary Contact, the simplest models assume homogeneous migration and homogeneous effective population size. 2N suffix = heterogeneous effective population size, 2M suffix = heterogeneous migration. Model with both suffix assumes that both effective population size and migration are heterogeneous. Model with a single suffix assumes that either migration or effective population size are heterogeneous. In one cases (Calif2 vs Thompson) the SC2N, SC2m and SC2N2m provided similar fit. For consistency the SC2N2m was considered for parameter estimates. Its parameter estimates were more consistent than those from either SC2N or SC2m. All tested models included the possibility for expansion or contraction of the daughter populations (not displayed here).
(TIF)

S4 Table Parameter estimates obtained under the best demographic model with ∂a∂i. Ne1 and Ne2, effective population size of the compared pair. m1 ← 2 and m2 ← 1, migration from population 2 to population 1 and migration from population 1 into population 2. me12 and me21, effective migration rate estimated in the most differentiated regions of the genome Ts: Split Time of the ancestral population in two population; Tsc: duration of the secondary contact P: proportion of the genome freely exchanged (1-P provides the proportion of the genome non-neutrally exchanged); Q: proportion of the genome with a reduced effective population size due to selection at linked sites; hrf = Hill-Robertson factor representing the reduction of Ne in the region Q with reduced Ne.
(TIF)

S5 Table Confidence Intervals obtained from the Godambe Information Matrix (GIM). (TIF)

S6 Table Confidence Intervals obtained from 100 dataset constructed with the coalescent simulator ms. (TIF)

S7 Table Parameter estimates and confidence intervals obtained from Fastsimcoal. (XLS)

S8 Table Tajjima’s D value and π_N_/π_S_ value observed for each locality. (TIF)

S9 Table Summary of deleterious variation by region. 1)Derived Allele Frequency (DAF) of deleterious mutation, after averaging by rivers and then by major regional group. 2) Count of deleterious mutations in each river and then averaged by major regional group. 3) Number of homozygous derived deleterious mutations by individual, after averaging by rivers and then by major regional group. 4) Number of heterozygous mutations by individuals, after averaging by rivers and then by major regional group 5) Total load of derived deleterious mutations by individuals, after averaging by rivers and then by major regional group.
(TIF)

S10 Table Results of Wilcoxon test (Mann-Whitney tests) for differences in derived allele frequencies among major groups for a sample of size 100. (TIF)

S11 Table Results of Wilcoxon test (Mann-Whitney tests) for differences in count of derived homozygous variants and total load among individuals in each region. (TIF)

S1 Text Details of Fastsimcoal analyses to infer a global scenario of divergence. The tested model is displayed in Fig 7 and S7 Table provide details of parameter estimates.
(PDF)

## References

[1] Provan J, Bennett KD. Phylogeographic insights into cryptic glacial refugia. Trends Ecol Evol (Amst). 2008;23: 564–571. 10.1016/j.tree.2008.06.010 18722689

[2] Hewitt GM. Post-glacial re-colonization of European biota. Biological Journal of the Linnean Society. 1999;68: 87–112. 10.1006/bijl.1999.0332

[3] Hewitt GM. Genetic consequences of climatic oscillations in the Quaternary. Philos Trans R Soc Lond, B, Biol Sci. 2004;359: 183–195; discussion 195. 10.1098/rstb.2003.1388 15101575 PMC1693318

[4] Bernatchez L, Wilson CC. Comparative phylogeography of Nearctic and Palearctic fishes. Molecular Ecology. 1998;7: 431–452. 10.1046/j.1365-294x.1998.00319.x

[5] Frankham R, Ballou JD, Briscoe DA. Introduction to Conservation Genetics by Richard Frankham. In: Cambridge Core [Internet]. Jan 2010 [cited 2 Jul 2019]. 10.1017/CBO9780511809002

[6] Funk WC, McKay JK, Hohenlohe PA, Allendorf FW. Harnessing genomics for delineating conservation units. Trends in Ecology & Evolution. 2012;27: 489–496. 10.1016/j.tree.2012.05.012 22727017 PMC4185076

[7] Simons YB, Turchin MC, Pritchard JK, Sella G. The deleterious mutation load is insensitive to recent population history. Nature Genetics. 2014;46: 220–224. 10.1038/ng.2896 24509481 PMC3953611

[8] Kirkpatrick M, Jarne P. The Effects of a Bottleneck on Inbreeding Depression and the Genetic Load. Am Nat. 2000;155: 154–167. 10.1086/303312 10686158

[9] Simons YB, Sella G. The impact of recent population history on the deleterious mutation load in humans and close evolutionary relatives. Curr Opin Genet Dev. 2016;41: 150–158. 10.1016/j.gde.2016.09.006 27744216 PMC5161708

[10] Bierne N, Gagnaire P-A, David P. The geography of introgression in a patchy environment and the thorn in the side of ecological speciation. Curr Zool. 2013;59: 72–86. 10.1093/czoolo/59.1.72

[11] Barton N, Bengtsson BO. The barrier to genetic exchange between hybridising populations. Heredity. 1986;57: 357 10.1038/hdy.1986.135 3804765

[12] Cruickshank TE, Hahn MW. Reanalysis suggests that genomic islands of speciation are due to reduced diversity, not reduced gene flow. Mol Ecol. 2014;23: 3133–3157. 10.1111/mec.12796 24845075

[13] Noor M a. F, Bennett SM. Islands of speciation or mirages in the desert? Examining the role of restricted recombination in maintaining species. Heredity (Edinb). 2009;103: 439–444. 10.1038/hdy.2009.151 19920849 PMC2809014

[14] Charlesworth B. The Effects of Deleterious Mutations on Evolution at Linked Sites. Genetics. 2012;190: 5–22. 10.1534/genetics.111.134288 22219506 PMC3249359

[15] Charlesworth B, Morgan MT, Charlesworth D. The effect of deleterious mutations on neutral molecular variation. Genetics. 1993;134: 1289–1303. 8375663 10.1093/genetics/134.4.1289 PMC1205596

[16] Pouyet F, Aeschbacher S, Thiéry A, Excoffier L. Background selection and biased gene conversion affect more than 95% of the human genome and bias demographic inferences. Veeramah K, Wittkopp PJ, Gronau I, editors. eLife. 2018;7: e36317 10.7554/eLife.36317 30125248 PMC6177262

[17] Roux C, Fraïsse C, Romiguier J, Anciaux Y, Galtier N, Bierne N. Shedding Light on the Grey Zone of Speciation along a Continuum of Genomic Divergence. PLOS Biology. 2016;14: e2000234 10.1371/journal.pbio.2000234 28027292 PMC5189939

[18] Comeron JM. Background selection as null hypothesis in population genomics: insights and challenges from Drosophila studies. Philos Trans R Soc Lond, B, Biol Sci. 2017;372 10.1098/rstb.2016.0471 29109230 PMC5698629

[19] Charlesworth D, Willis JH. The genetics of inbreeding depression. Nat Rev Genet. 2009;10: 783–796. 10.1038/nrg2664 19834483

[20] Kyriazis C, Wayne RK, Lohmueller KE. High genetic diversity can contribute to extinction in small populations | bioRxiv. [cited 10 Mar 2020]. Available: https://www.biorxiv.org/content/10.1101/678524v1

[21] Robinson JA, Räikkönen J, Vucetich LM, Vucetich JA, Peterson RO, Lohmueller KE, et al Genomic signatures of extensive inbreeding in Isle Royale wolves, a population on the threshold of extinction. Sci Adv. 2019;5: eaau0757 10.1126/sciadv.aau0757 31149628 PMC6541468

[22] Robinson JA, Ortega-Del Vecchyo D, Fan Z, Kim BY, vonHoldt BM, Marsden CD, et al Genomic Flatlining in the Endangered Island Fox. Curr Biol. 2016;26: 1183–1189. 10.1016/j.cub.2016.02.062 27112291

[23] Abascal F, Corvelo A, Cruz F, Villanueva-Cañas JL, Vlasova A, Marcet-Houben M, et al Extreme genomic erosion after recurrent demographic bottlenecks in the highly endangered Iberian lynx. Genome Biol. 2016;17: 251 10.1186/s13059-016-1090-1 27964752 PMC5155386

[24] Dobrynin P, Liu S, Tamazian G, Xiong Z, Yurchenko AA, Krasheninnikova K, et al Genomic legacy of the African cheetah, Acinonyx jubatus. Genome Biol. 2015;16: 277 10.1186/s13059-015-0837-4 26653294 PMC4676127

[25] Yang Y, Ma T, Wang Z, Lu Z, Li Y, Fu C, et al Genomic effects of population collapse in a critically endangered ironwood tree Ostrya rehderiana. Nat Commun. 2018;9: 5449 10.1038/s41467-018-07913-4 30575743 PMC6303402

[26] Xue Y, Prado-Martinez J, Sudmant PH, Narasimhan V, Ayub Q, Szpak M, et al Mountain gorilla genomes reveal the impact of long-term population decline and inbreeding. Science. 2015;348: 242–245. 10.1126/science.aaa3952 25859046 PMC4668944

[27] Grossen C, Guillaume F, Keller LF, Croll D. Accumulation and purging of deleterious mutations through severe bottlenecks in ibex. bioRxiv. 2019; 605147. 10.1101/605147 PMC7035315 32081890

[28] Henn BM, Botigué LR, Peischl S, Dupanloup I, Lipatov M, Maples BK, et al Distance from sub-Saharan Africa predicts mutational load in diverse human genomes. PNAS. 2016;113: E440–E449. 10.1073/pnas.1510805112 26712023 PMC4743782

[29] Henn BM, Botigué LR, Bustamante CD, Clark AG, Gravel S. Estimating the mutation load in human genomes. Nat Rev Genet. 2015;16: 333–343. 10.1038/nrg3931 25963372 PMC4959039

[30] Krkosek M, Ford JS, Morton A, Lele S, Myers RA, Lewis MA. Declining wild salmon populations in relation to parasites from farm salmon. Science. 2007;318: 1772–1775. 10.1126/science.1148744 18079401

[31] Irvine JR, Fukuwaka M. Pacific salmon abundance trends and climate change. ICES J Mar Sci. 2011;68: 1122–1130. 10.1093/icesjms/fsq199

[32] Gustafson RG, Waples RS, Myers JM, Weitkamp LA, Bryant GJ, Johnson OW, et al Pacific Salmon Extinctions: Quantifying Lost and Remaining Diversity. Conservation Biology. 2007;21: 1009–1020. 10.1111/j.1523-1739.2007.00693.x 17650251

[33] Smith CT, Nelson RJ, Wood CC, Koop BF. Glacial biogeography of North American coho salmon (Oncorhynchus kisutch). Mol Ecol. 2001;10: 2775–2785. 10.1046/j.1365-294x.2001.t01-1-01405.x 11903891

[34] Beacham TD, Wetklo M, Deng L, MacConnachie C. Coho Salmon Population Structure in North America Determined from Microsatellites. Transactions of the American Fisheries Society. 2011;140: 253–270. 10.1080/00028487.2011.558782

[35] McPhail JD, Lindsey CC. Freshwater fishes of northwestern Canada and Alaska. Fisheries Research Board of Canada: available by mail from the Queen’s Printer; 1970.

[36] UBC Press | Pacific Salmon Life Histories, By Cornelis Groot, Leo Margolis and Leo Margolis. In: UBC Press [Internet]. [cited 1 Jul 2019]. Available: https://www.ubcpress.ca/pacific-salmon-life-histories

[37] Hocutt CH, Wiley EO, editors. The Zoogeography of North American Freshwater Fishes. 1 edition New York: Wiley-Interscience; 1986.

[38] Mee JA, Moore J-S. The ecological and evolutionary implications of microrefugia. Journal of Biogeography. 2014;41: 837–841. 10.1111/jbi.12254

[39] Warner BG, Mathewes RW, Clague JJ. Ice-free conditions on the queen charlotte islands, british columbia, at the height of late wisconsin glaciation. Science. 1982;218: 675–677. 10.1126/science.218.4573.675 17791586

[40] Li JZ, Devin AM, Tang H, Southwick AM, Casto AM, Ramachandran S, et al Worldwide Human Relationships Inferred from Genome-Wide Patterns of Variation. Science. 2008;319: 1100–1104. 10.1126/science.1153717 18292342

[41] Petit RJ, Aguinagalde I, Beaulieu J-L de, Bittkau C, Brewer S, Cheddadi R, et al Glacial Refugia: Hotspots But Not Melting Pots of Genetic Diversity. Science. 2003;300: 1563–1565. 10.1126/science.1083264 12791991

[42] Weir BS, Goudet J. A Unified Characterization of Population Structure and Relatedness. Genetics. 2017;206: 2085–2103. 10.1534/genetics.116.198424 28550018 PMC5560808

[43] Cubry P, Vigouroux Y, François O. The Empirical Distribution of Singletons for Geographic Samples of DNA Sequences. Front Genet. 2017;8 10.3389/fgene.2017.00139 29033977 PMC5627571

[44] Gilbert-Horvath EA, Pipal KA, Spence BC, Williams TH, Garza JC. Hierarchical Phylogeographic Structure of Coho Salmon in California. Transactions of the American Fisheries Society. 2016;145: 1122–1138. 10.1080/00028487.2016.1201003

[45] Williams TH, Lindley ST, Spence BC, Boughton DA. STATUS REVIEW UPDATE FOR PACIFIC SALMON AND STEELHEAD LISTED UNDER THE ENDANGERED SPECIES ACT: SOUTHWEST.: 106.

[46] Weir BS, Cockerham CC. Estimating F-Statistics for the Analysis of Population Structure. Evolution. 1984;38: 1358–1370. 10.1111/j.1558-5646.1984.tb05657.x 28563791

[47] Ward RD, Woodwark M, Skibinski DOF. A comparison of genetic diversity levels in marine, freshwater, and anadromous fishes. Journal of Fish Biology. 1994;44: 213–232. 10.1111/j.1095-8649.1994.tb01200.x

[48] Patterson N, Price AL, Reich D. Population structure and eigenanalysis. PLoS Genet. 2006;2: e190 10.1371/journal.pgen.0020190 17194218 PMC1713260

[49] Novembre J, Stephens M. Interpreting principal component analyses of spatial population genetic variation. Nat Genet. 2008;40: 646–649. 10.1038/ng.139 18425127 PMC3989108

[50] Frichot E, François O. LEA: An R package for landscape and ecological association studies. Methods in Ecology and Evolution. 2015;6: 925–929. 10.1111/2041-210X.12382

[51] Meirmans PG. The trouble with isolation by distance. Mol Ecol. 2012;21: 2839–2846. 10.1111/j.1365-294X.2012.05578.x 22574758

[52] Pickrell JK, Pritchard JK. Inference of Population Splits and Mixtures from Genome-Wide Allele Frequency Data. PLOS Genetics. 2012;8: e1002967 10.1371/journal.pgen.1002967 23166502 PMC3499260

[53] Gutenkunst RN, Hernandez RD, Williamson SH, Bustamante CD. Inferring the Joint Demographic History of Multiple Populations from Multidimensional SNP Frequency Data. PLOS Genetics. 2009;5: e1000695 10.1371/journal.pgen.1000695 19851460 PMC2760211

[54] Excoffier L, Dupanloup I, Huerta-Sánchez E, Sousa VC, Foll M. Robust Demographic Inference from Genomic and SNP Data. PLOS Genetics. 2013;9: e1003905 10.1371/journal.pgen.1003905 24204310 PMC3812088

[55] Nicolas Alcala, Vuilleumier Séverine. Turnover and accumulation of genetic diversity across large time-scale cycles of isolation and connection of populations. Proceedings of the Royal Society B: Biological Sciences. 2014;281: 20141369 10.1098/rspb.2014.1369 25253456 PMC4211446

[56] COSEWIC assessment and status report on the coho salmon Oncorhynchus kisutch (Interior Fraser population) in Canada—Species at Risk Public Registry. [cited 1 Jul 2019]. Available: https://wildlife-species.canada.ca/species-risk-registry/document/default_e.cfm?documentID=105

[57] J. Wang, Personal Communication.

[58] Lapierre M, Lambert A, Achaz G. Accuracy of Demographic Inferences from the Site Frequency Spectrum: The Case of the Yoruba Population. Genetics. 2017;206: 439–449. 10.1534/genetics.116.192708 28341655 PMC5419487

[59] Baharian S, Gravel S. On the decidability of population size histories from finite allele frequency spectra. Theoretical Population Biology. 2018;120: 42–51. 10.1016/j.tpb.2017.12.008 29305873

[60] Terhorst J, Song YS. Fundamental limits on the accuracy of demographic inference based on the sample frequency spectrum. PNAS. 2015;112: 7677–7682. 10.1073/pnas.1503717112 26056264 PMC4485089

[61] Quinn TP. Homing and Straying in Pacific Salmon In: McCleave JD, Arnold GP, Dodson JJ, Neill WH, editors. Mechanisms of Migration in Fishes. Boston, MA: Springer US; 1984 pp. 357–362. 10.1007/978-1-4613-2763-9_21

[62] Ewing GB, Jensen JD. The consequences of not accounting for background selection in demographic inference. Molecular Ecology. 2016;25: 135–141. 10.1111/mec.13390 26394805

[63] McVean G, Awadalla P, Fearnhead P. A Coalescent-Based Method for Detecting and Estimating Recombination From Gene Sequences. Genetics. 2002;160: 1231–1241. 11901136 10.1093/genetics/160.3.1231 PMC1462015

[64] data available on github at https://github.com/QuentinRougemont/coho_salmon_recomb/

[65] Peischl S, Dupanloup I, Kirkpatrick M, Excoffier L. On the accumulation of deleterious mutations during range expansions. Molecular Ecology. 2013;22: 5972–5982. 10.1111/mec.12524 24102784

[66] Peischl S, Dupanloup I, Foucal A, Jomphe M, Bruat V, Grenier J-C, et al Relaxed Selection During a Recent Human Expansion. Genetics. 2018;208: 763–777. 10.1534/genetics.117.300551 29187508 PMC5788536

[67] Peischl S, Excoffier L. Expansion load: recessive mutations and the role of standing genetic variation. Molecular Ecology. 2015;24: 2084–2094. 10.1111/mec.13154 25786336

[68] Chen J, Glémin S, Lascoux M. Genetic Diversity and the Efficacy of Purifying Selection across Plant and Animal Species, Molecular Biology and Evolution, 2017, 34;6,1417–1428, 10.1093/molbev/msx088 28333215

[69] Kim BY, Huber CD, Lohmueller KE. Deleterious variation shapes the genomic landscape of introgression. PLOS Genetics. 2018;14: e1007741 10.1371/journal.pgen.1007741 30346959 PMC6233928

[70] Choi Y, Sims GE, Murphy S, Miller JR, Chan AP. Predicting the functional effect of amino acid substitutions and indels. PLoS ONE. 2012;7: e46688 10.1371/journal.pone.0046688 23056405 PMC3466303

[71] Christensen KA, Leong JS, Sakhrani D, Biagi CA, Minkley DR, Withler RE, et al Chinook salmon (Oncorhynchus tshawytscha) genome and transcriptome. PLOS ONE. 2018;13: e0195461 10.1371/journal.pone.0195461 29621340 PMC5886536

[72] Yáñez JM, Naswa S, López ME, Bassini L, Correa K, Gilbey J, et al Genomewide single nucleotide polymorphism discovery in Atlantic salmon (Salmo salar): validation in wild and farmed American and European populations. Mol Ecol Resour. 2016;16: 1002–1011. 10.1111/1755-0998.12503 26849107

[73] Zhou Y, Massonnet M, Sanjak JS, Cantu D, Gaut BS. Evolutionary genomics of grape (Vitis vinifera ssp. vinifera) domestication. PNAS. 2017;114: 11715–11720. 10.1073/pnas.1709257114 29042518 PMC5676911

[74] Nevado B, Wong ELY, Osborne OG, Filatov DA. Adaptive Evolution Is Common in Rapid Evolutionary Radiations. Current Biology. 2019;29: 3081–3086.e5. 10.1016/j.cub.2019.07.059 31495580

[75] Willi Y, Fracassetti M, Zoller S, Van Buskirk J. Accumulation of Mutational Load at the Edges of a Species Range. Mol Biol Evol. 2018;35: 781–791. 10.1093/molbev/msy003 29346601

[76] González-Martínez SC, Ridout K, Pannell JR. Range Expansion Compromises Adaptive Evolution in an Outcrossing Plant. Current Biology. 2017;27: 2544–2551.e4. 10.1016/j.cub.2017.07.007 28803874

[77] Bosshard L, Dupanloup I, Tenaillon O, Bruggmann R, Ackermann M, Peischl S, et al Accumulation of Deleterious Mutations During Bacterial Range Expansions. Genetics. 2017;207: 669–684. 10.1534/genetics.117.300144 28821588 PMC5629331

[78] Lohmueller KE. The distribution of deleterious genetic variation in human populations. Curr Opin Genet Dev. 2014;29: 139–146. 10.1016/j.gde.2014.09.005 25461617

[79] Moore J-S, Harris LN, Le Luyer J, Sutherland BJG, Rougemont Q, Tallman RF, et al Genomics and telemetry suggest a role for migration harshness in determining overwintering habitat choice, but not gene flow, in anadromous Arctic Char. Mol Ecol. 2017;26: 6784–6800. 10.1111/mec.14393 29087005

[80] Li H. Aligning sequence reads, clone sequences and assembly contigs with BWA-MEM. arXiv:13033997 [q-bio]. 2013 [cited 3 Jul 2019]. Available: http://arxiv.org/abs/1303.3997

[81] Li H, Handsaker B, Wysoker A, Fennell T, Ruan J, Homer N, et al The Sequence Alignment/Map format and SAMtools. Bioinformatics. 2009;25: 2078–2079. 10.1093/bioinformatics/btp352 19505943 PMC2723002

[82] Catchen JM, Amores A, Hohenlohe P, Cresko W, Postlethwait JH. Stacks: Building and Genotyping Loci De Novo From Short-Read Sequences. G3: Genes, Genomes, Genetics. 2011;1: 171–182. 10.1534/g3.111.000240 22384329 PMC3276136

[83] Korneliussen TS, Albrechtsen A, Nielsen R. ANGSD: Analysis of Next Generation Sequencing Data. BMC Bioinformatics. 2014;15: 356 10.1186/s12859-014-0356-4 25420514 PMC4248462

[84] Warmuth VM, Ellegren H. Genotype-free estimation of allele frequencies reduces bias and improves demographic inference from RADSeq data. Molecular Ecology Resources. 2019;19: 586–596. 10.1111/1755-0998.12990 30633448

[85] Danecek P, Auton A, Abecasis G, Albers CA, Banks E, DePristo MA, et al The variant call format and VCFtools. Bioinformatics. 2011;27: 2156–2158. 10.1093/bioinformatics/btr330 21653522 PMC3137218

[86] Goudet J. hierfstat, a package for r to compute and test hierarchical F-statistics. Molecular Ecology Notes. 2005;5: 184–186. 10.1111/j.1471-8286.2004.00828.x

[87] Benjamini Y., and Hochberg Y. (1995). Controlling the false discovery rate: a practical and powerful approach to multiple testing. _Journal of the Royal Statistical Society Series B, *57*, 289–300. <URL: http://www.jstor.org/stable/2346101>.

[88] Alexander DH, Novembre J, Lange K. Fast model-based estimation of ancestry in unrelated individuals. Genome Res. 2009;19: 1655–1664. 10.1101/gr.094052.109 19648217 PMC2752134

[89] Dray S, Dufour A (2007). “The ade4 Package: Implementing the Duality Diagram for Ecologists.” *Journal of Statistical Software*, 22(4), 1–20. 10.18637/jss.v022.i04

[90] Wickham H. ggplot2: Elegant Graphics for Data Analysis. New York: Springer-Verlag; 2009 Available: https://www.springer.com/gp/book/9780387981413

[91] Rougemont Q, Gagnaire P-A, Perrier C, Genthon C, Besnard A-L, Launey S, et al Inferring the demographic history underlying parallel genomic divergence among pairs of parasitic and nonparasitic lamprey ecotypes. Molecular Ecology. 2017;26: 142–162. 10.1111/mec.13664 27105132

[92] Hudson RR. Generating samples under a Wright-Fisher neutral model of genetic variation. Bioinformatics. 2002;18: 337–338. 10.1093/bioinformatics/18.2.337 11847089

[93] Leroy T, Anselmetti A, Tilak MK, Bérard S, Csukonyi L, Gabrielli M, Scornavacca C, Milá B, Thébaud C and Nabholz B. A bird’s white-eye view on neo-sex chromosome evolution. bioRxiv. 2019; 505610, ver. 4 peer-reviewed and recommended by PCI Evolutionary Biology. 10.1101/505610

[94] Government of Canada NRC. GEOSCAN Search Results: Fastlink [Internet]. 7 12 2015 [cited 26 Jul 2019]. Available: https://geoscan.nrcan.gc.ca/starweb/geoscan/servlet.starweb?path=geoscan/fulle.web&search1=R=214399

[95] https://www.naturalearthdata.com/downloads/10m-physical-vectors/ last access 10-05-2020.

[96] Clark PU, Dyke AS, Shakun JD, Carlson AE, Clark J, Wohlfarth B, et al The Last Glacial Maximum. Science. 2009;325: 710–714. 10.1126/science.1172873 19661421

[97] Elias SA, Brigham-Grette J. GLACIATIONS | Late Pleistocene Events in Beringia In: Elias SA, editor. Encyclopedia of Quaternary Science. Oxford: Elsevier; 2007 pp. 1057–1066. 10.1016/B0-44-452747-8/00132-0

