# Correction: Demographic history shaped geographical patterns of deleterious mutation load in a broadly distributed Pacific Salmon

**DOI:** 10.1371/journal.pgen.1009397

**Published:** 2021-02-18

**Authors:** Quentin Rougemont, Jean-Sébastien Moore, Thibault Leroy, Eric Normandeau, Eric B. Rondeau, Ruth E. Withler, Donald M. Van Doornik, Penelope A. Crane, Kerry A. Naish, John Carlos Garza, Terry D. Beacham, Ben F. Koop, Louis Bernatchez

Fig 2 is incorrectly cropped, removing four points in the dataset. The authors have provided a corrected version here.

**Fig 2:**
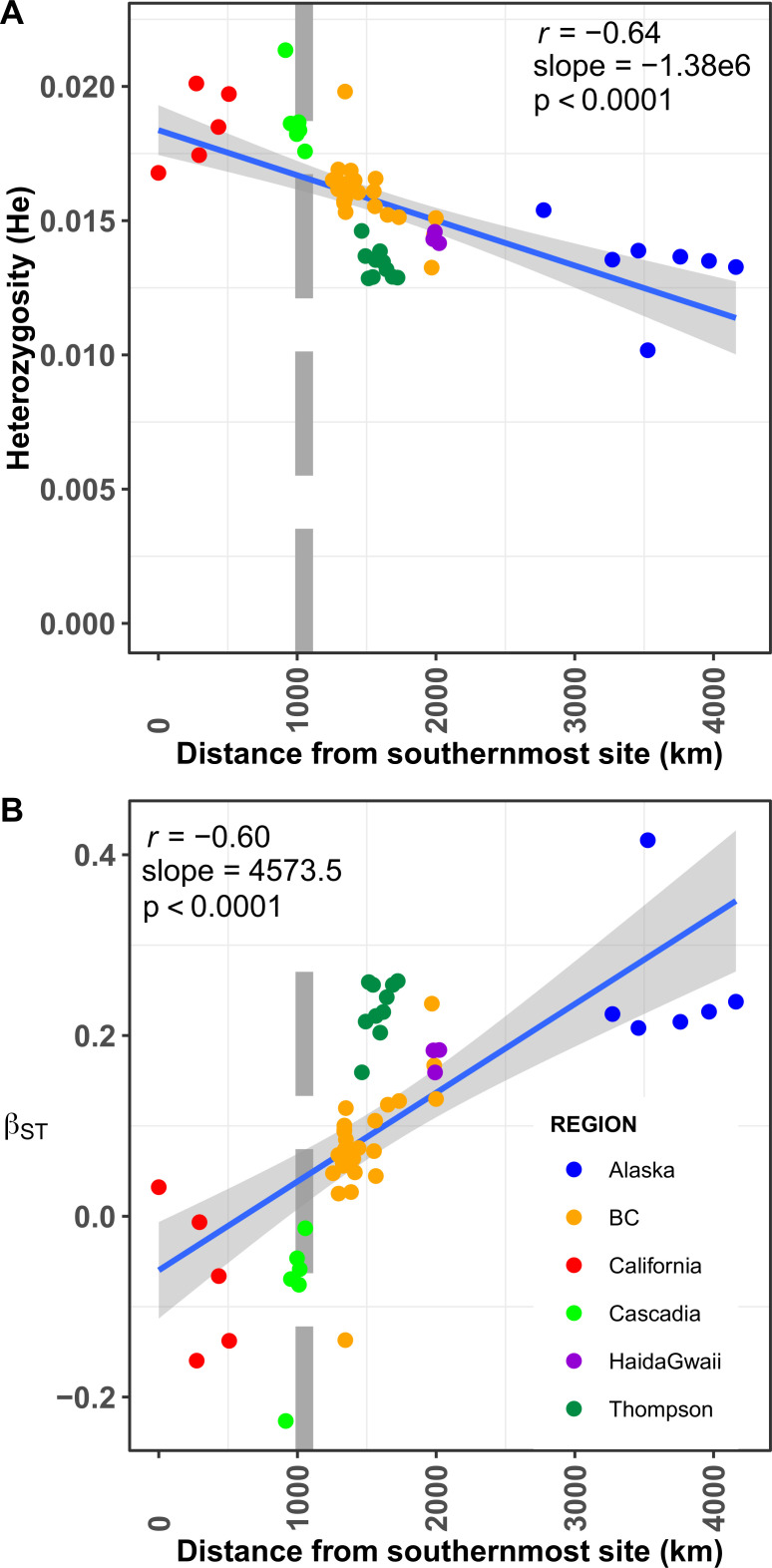
Genetic diversity and differentiation. A) Linear relationship between expected heterozygosity and distance from the southernmost population located in California. B) Linear increase in genetic differentiation as measured by β_ST_ as a function of the distance from the southernmost population located in California. Negative values indicate the most likely ancestral population. The relationship in A and B was tested using linear models. The grey vertical bar in panel A and B indicates the approximate location of the southern limit of the ice-sheet at the end of the last glacial maximum. The coefficient of correlation (r) is indicated for each plot. The grey shaded area along the regression line corresponds to the 95% confidence level interval obtained from the linear models applied to each dataset.

## References

[1] Rougemont Q, Moore J-S, Leroy T, Normandeau E, Rondeau EB, Withler RE, et al (2020) Demographic history shaped geographical patterns of deleterious mutation load in a broadly distributed Pacific Salmon. PLoS Genet 16(8): e1008348 10.1371/journal.pgen.1008348 32845885 PMC7478589

