# Supplementary figures and images for: Demographic history shaped geographical patterns of deleterious mutation load in a broadly distributed Pacific Salmon
Source: PLoS Genet. 2020 Aug 26;16(8):e1008348. doi: 10.1371/journal.pgen.1008348 (PMC7478589; doi:10.1371/journal.pgen.1008348)

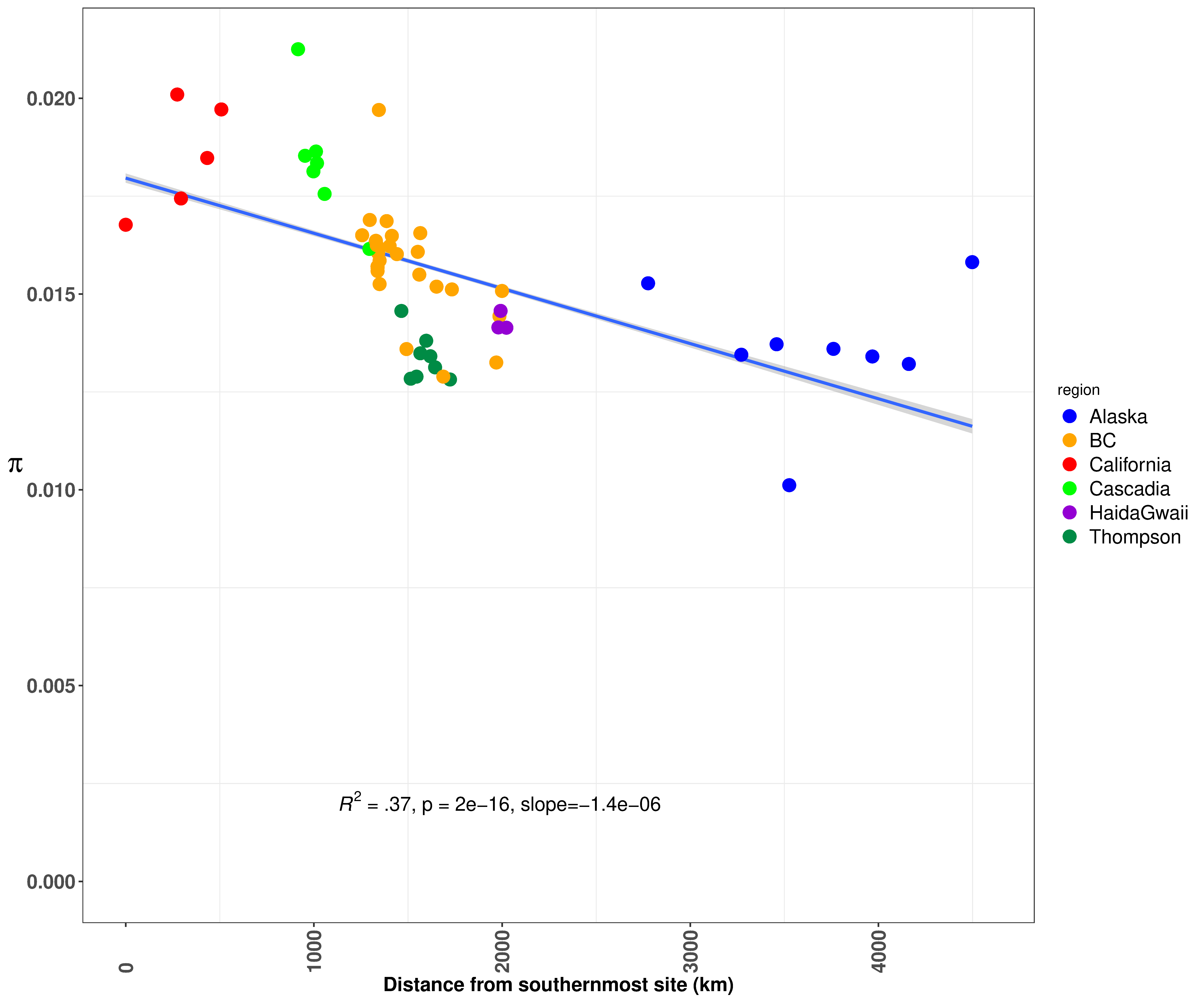

Supplement: S1 Fig — Each points represents a sample site and is colored by region. (TIF) [file pgen.1008348.s001.tif]

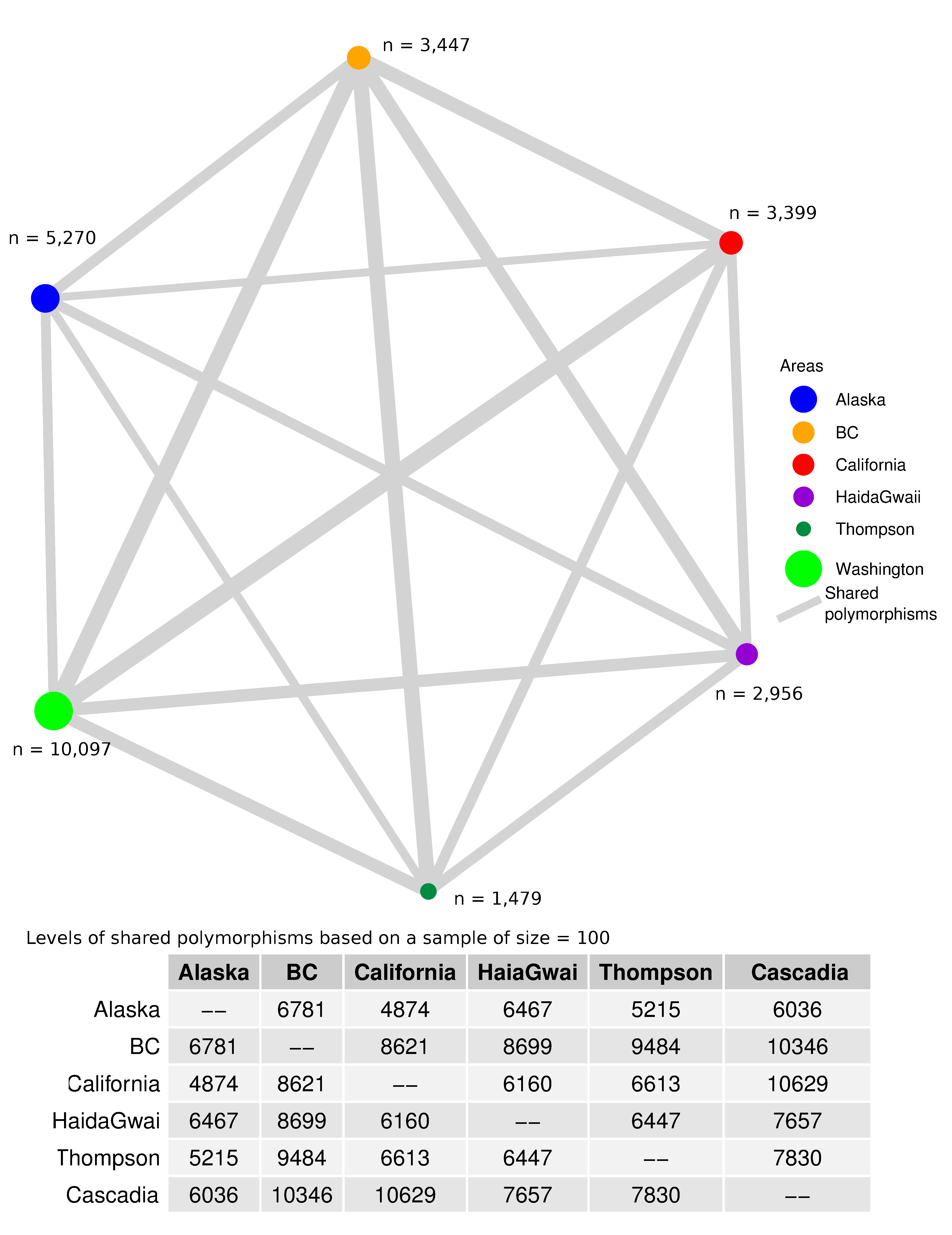

Supplement: S2 Fig — The branch (grey) represent shared polymorphism between sample site and are proportional to levels of sharing. Each point represents the number of private polymorphism and is colored by region. Computation were based on a sample of size 100 in each region to enable comparison. Regional groups were chosen based on the literature regarding expected ancestral refugia. (TIF) [file pgen.1008348.s002.tif]

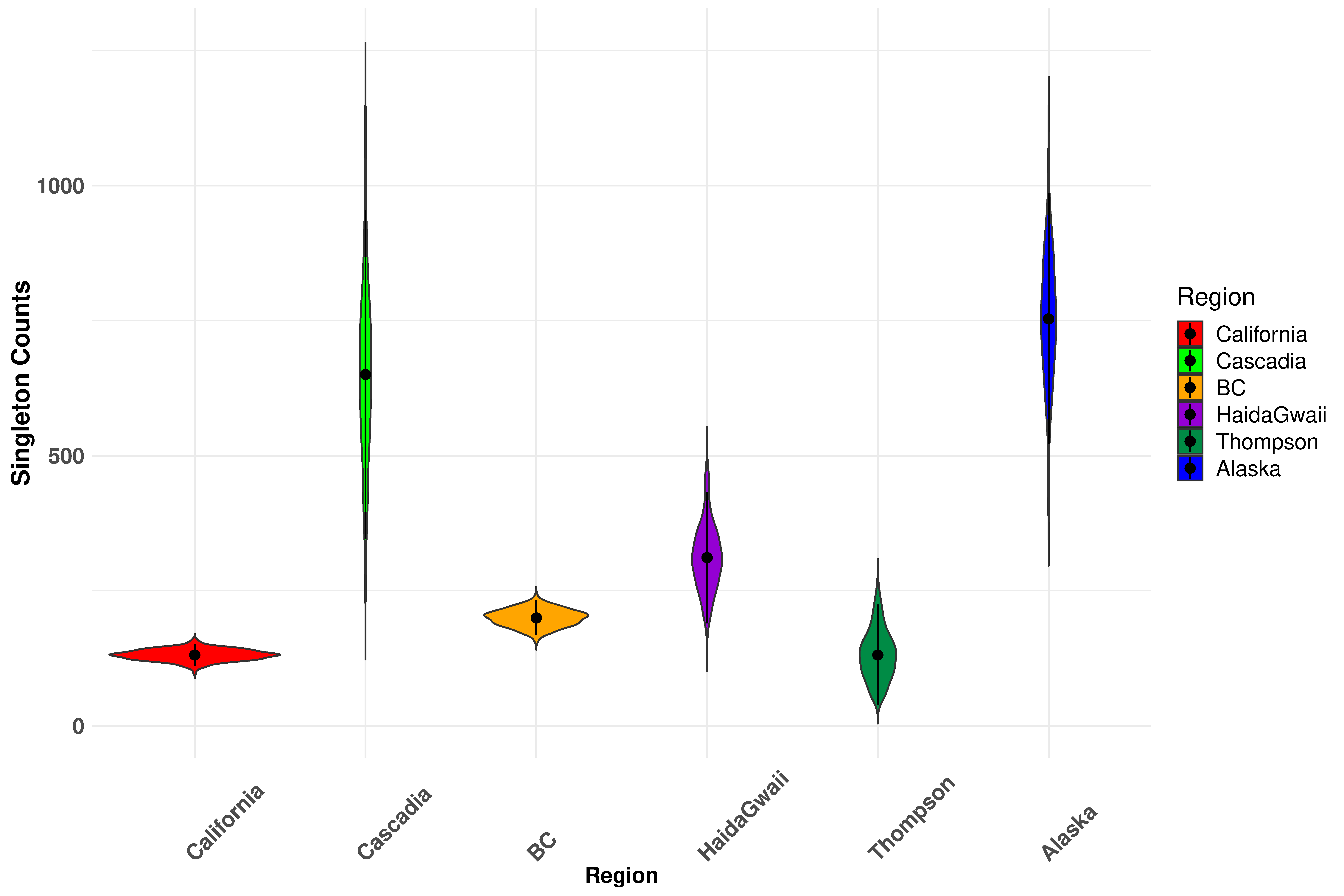

Supplement: S3 Fig — Shown is the distribution observed across 200 dataset obtained by randomly sampling individuals across populations. Black dots with errors bars represent the mean ±1 standard deviation. (TIF) [file pgen.1008348.s003.tif]

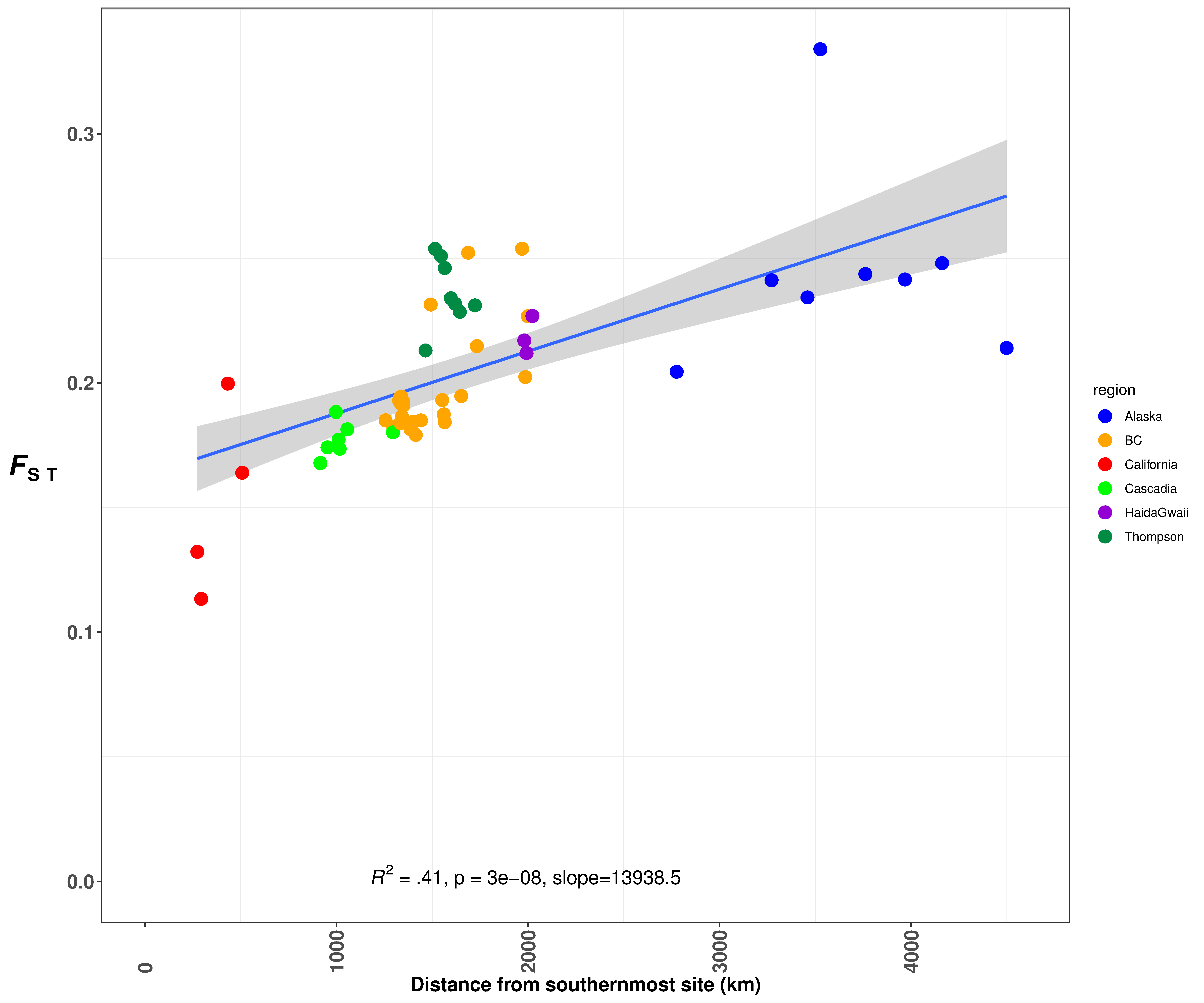

Supplement: S4 Fig — Increasing FST as a function of the distance to the southernmost site. Each point represents a sample site and is coloured by region. The FST was computed between the southernmost site and all other remaining sites. (TIF) [file pgen.1008348.s004.tif]

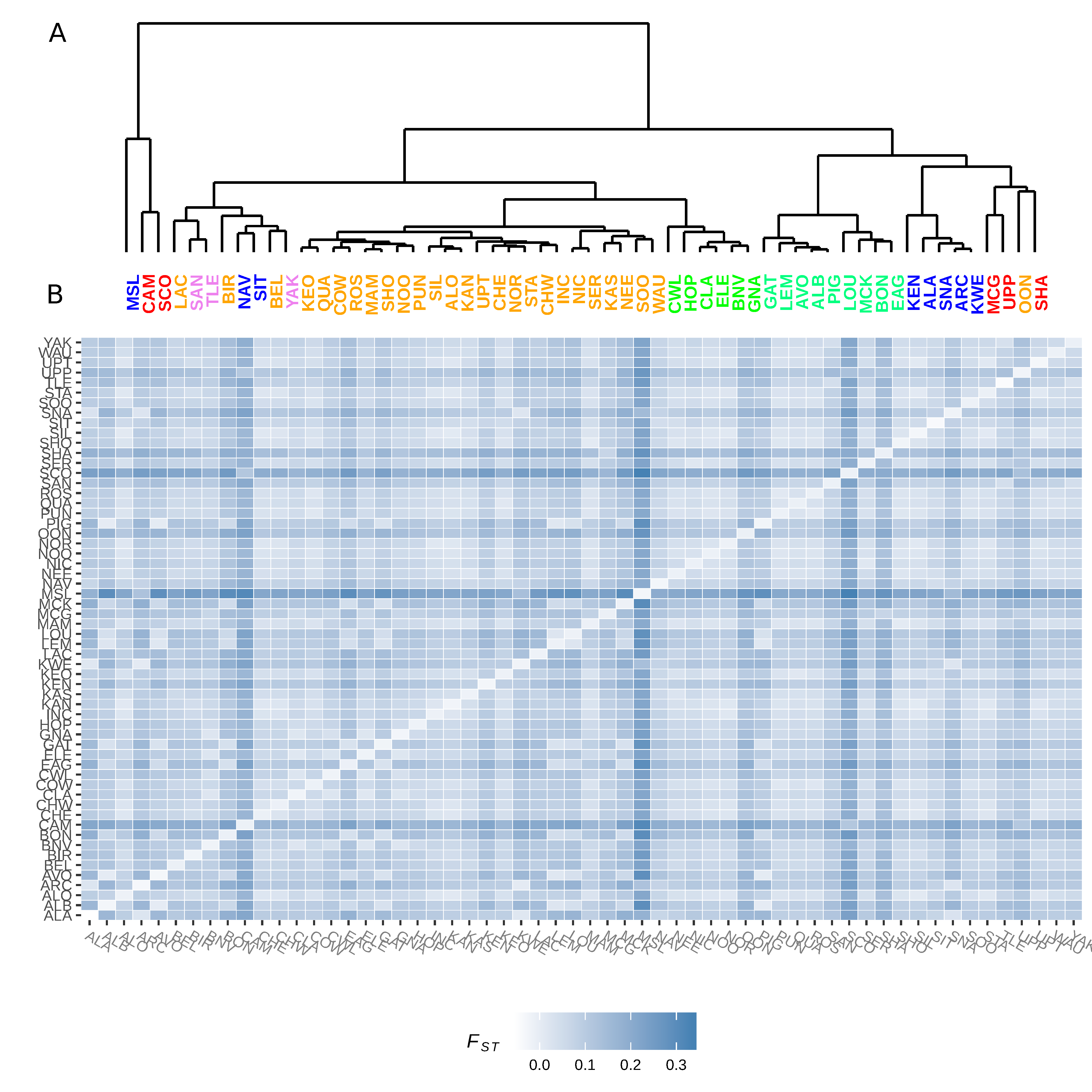

Supplement: S5 Fig — A. FST-based Hierarchical tree depicting relationship among samples. Colors represent the major region. B. Heatmap of FST values among samples ordered from North to South on the X and Y-axis. (TIF) [file pgen.1008348.s005.tif]

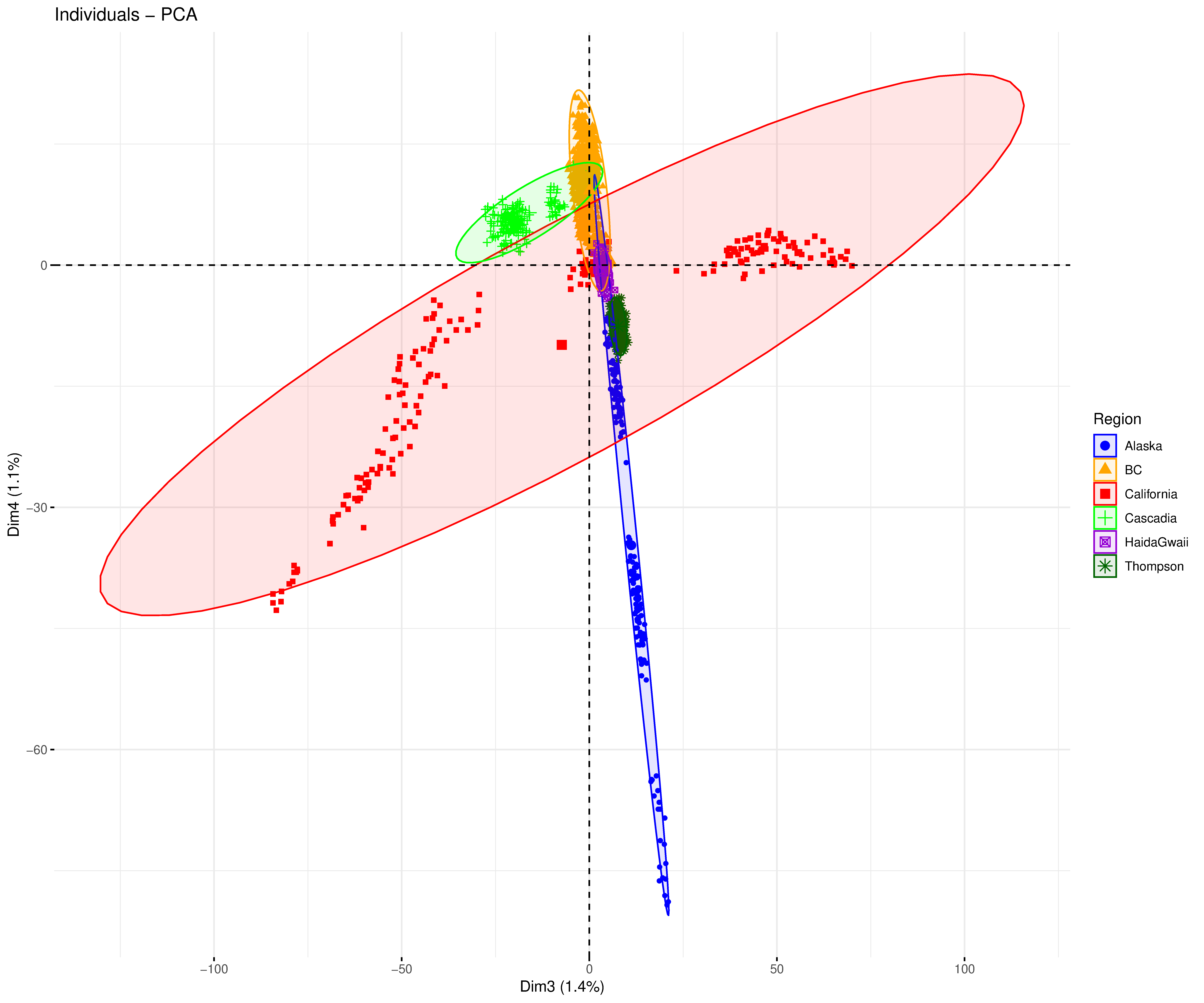

Supplement: S6 Fig — The Axis 3 and axis 4 are displayed. (TIF) [file pgen.1008348.s006.tif]

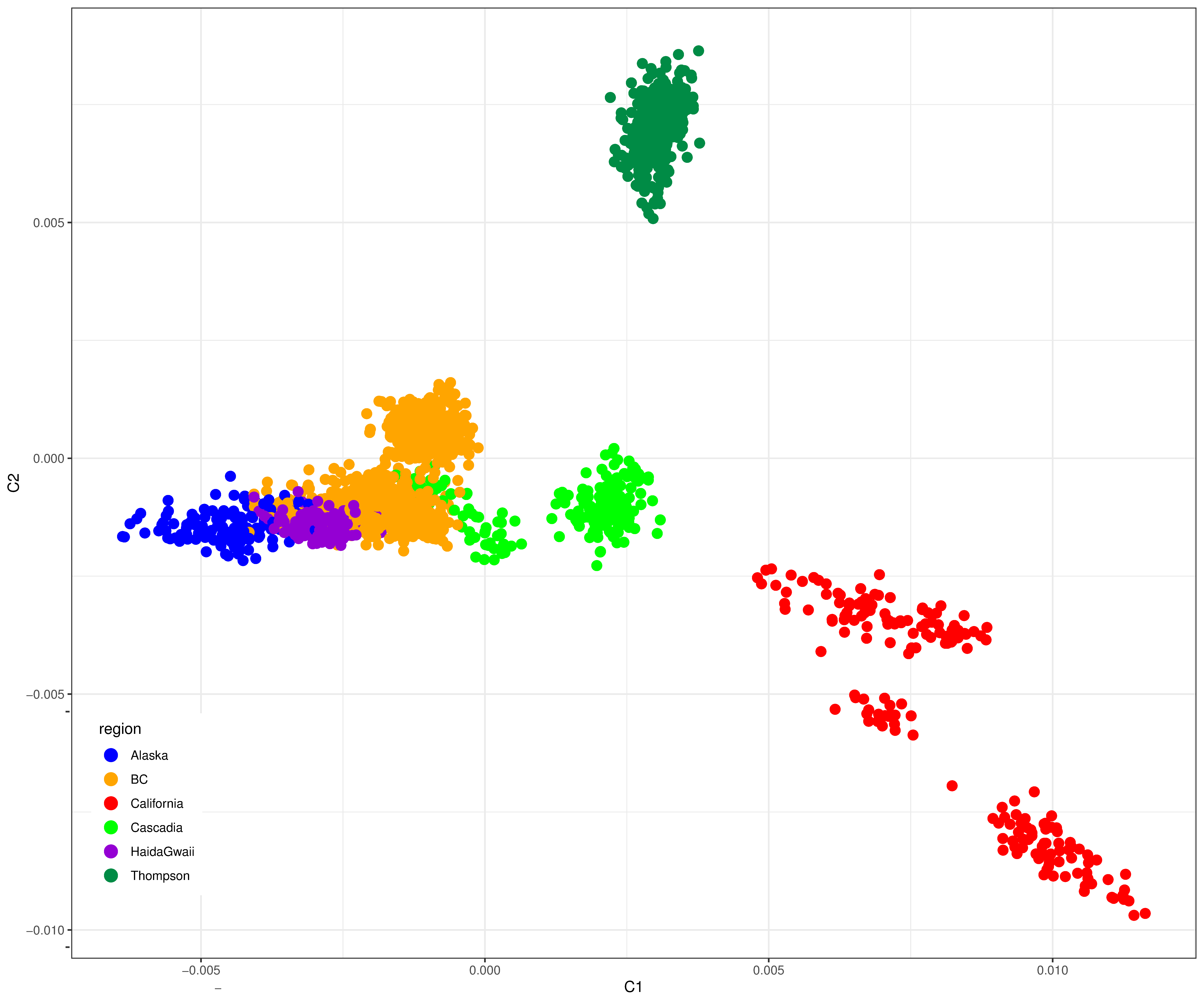

Supplement: S7 Fig — Each point represents an individual site and is colored by region. (TIF) [file pgen.1008348.s007.tif]

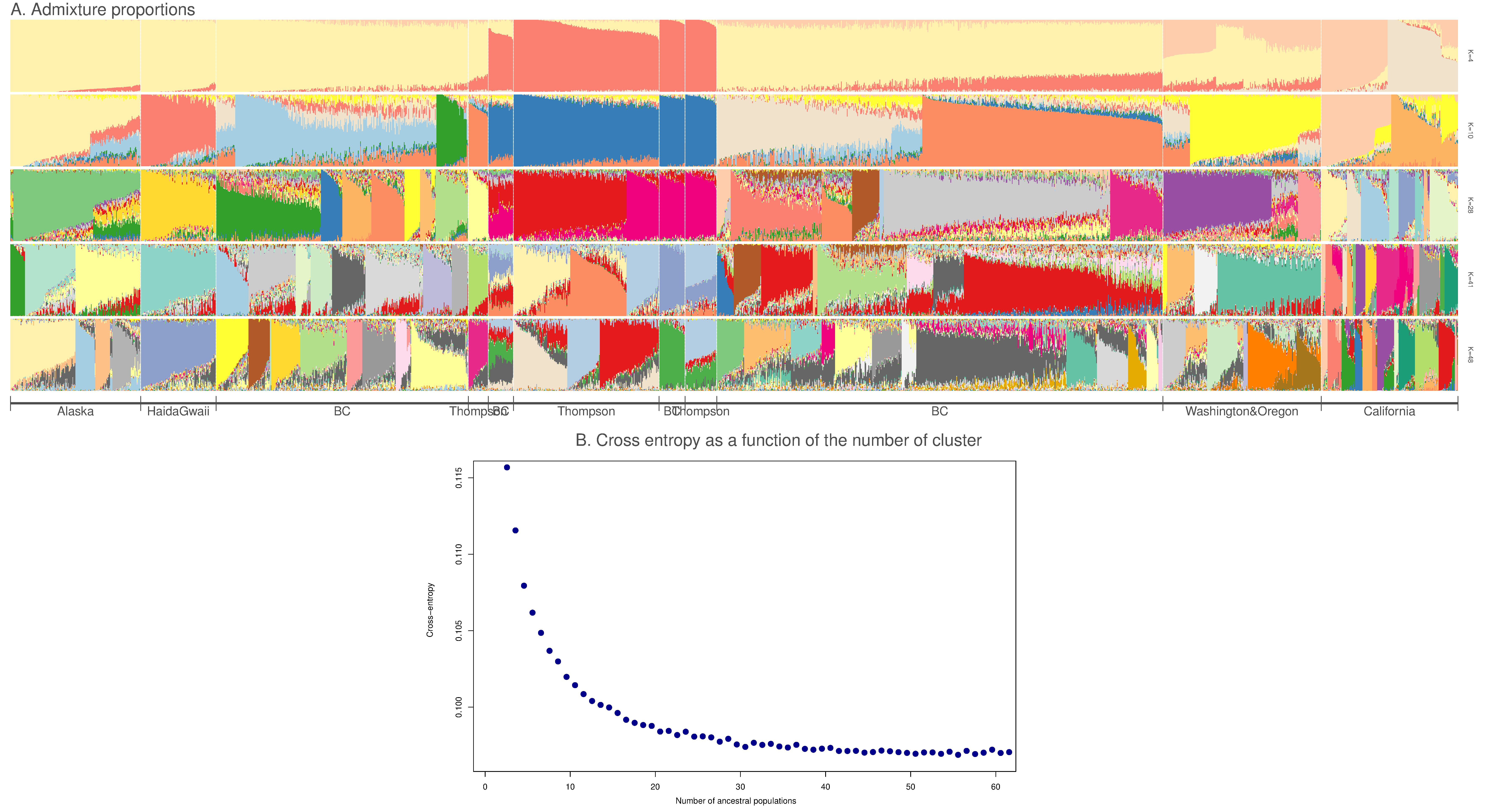

Supplement: S8 Fig — A. Admixture Barplot obtained from LEA for various K-values. B. Progressive decrease of LEA cross-entropy criterion. Lower cross-entropy values indicates the number of cluster compatible with the data (here from 30 to 60). (TIF) [file pgen.1008348.s008.tif]

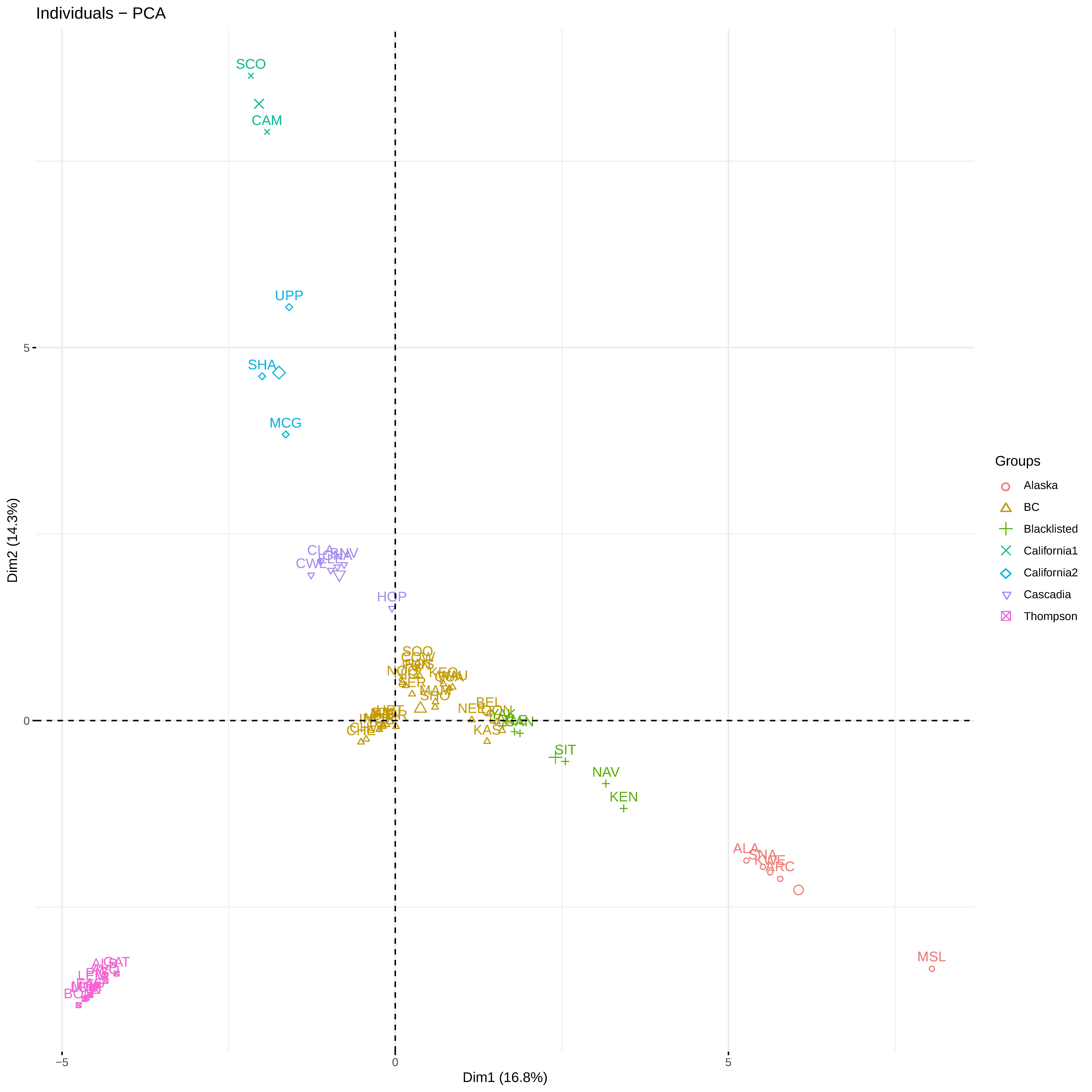

Supplement: S10 Fig — The site indicated as “blacklisted” are sites that were not included in the ∂a∂i analyses. These corresponds to potentially admixed sites between BC and Alaska and display a reduced number of individuals to constitute a coherent unit for demographic comparison. (TIF) [file pgen.1008348.s010.tif]

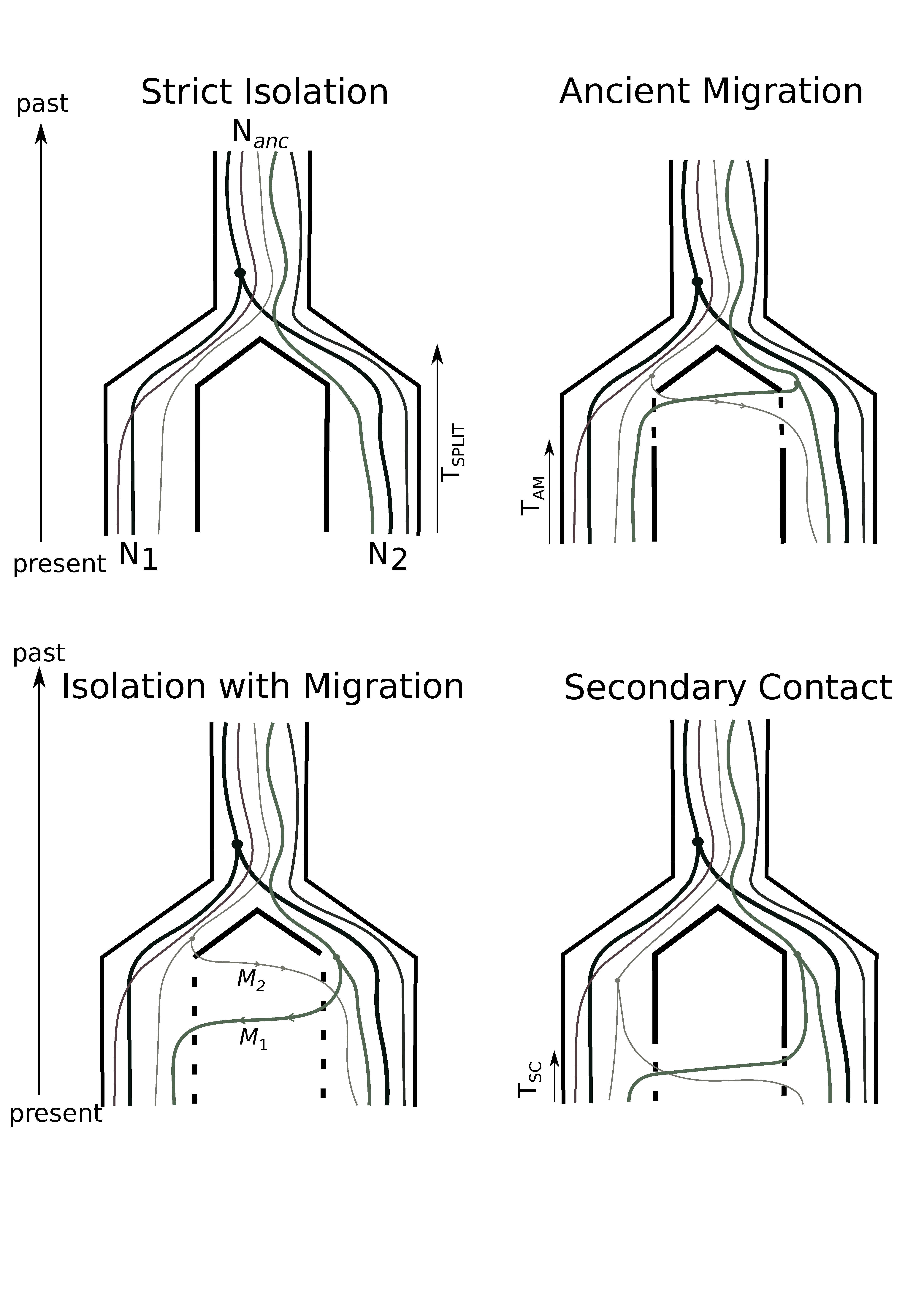

Supplement: S11 Fig — Strict Isolation (SI), Isolation with constant Migration (IM), Ancient Migration (AM) and Secondary Contact (SC). The models shared the following parameters: Tsplit: number of generation of divergence (backwards in time). Nanc, N1, N2: effective population size of the ancestral population, of the first and second daughter population. M1 and M2 represent the effective migration rates per generation that is (M = 2.Nref.m) with m the proportion of population made of migrants from the other population and Nref the size of the reference population. Tsc is the number of generations since gene flow started (secondary contact) after a period of isolation. Tam is the number of generations since the two populations have diverged without gene flow until present. Each model is declined in alternative version allowing homogeneous or heterogeneous effective size and homogeneous or heterogeneous gene flow to account for the effect of linked selection (affecting Ne) and barrier to gene flow (affecting m) respective. (TIF) [file pgen.1008348.s011.tif]

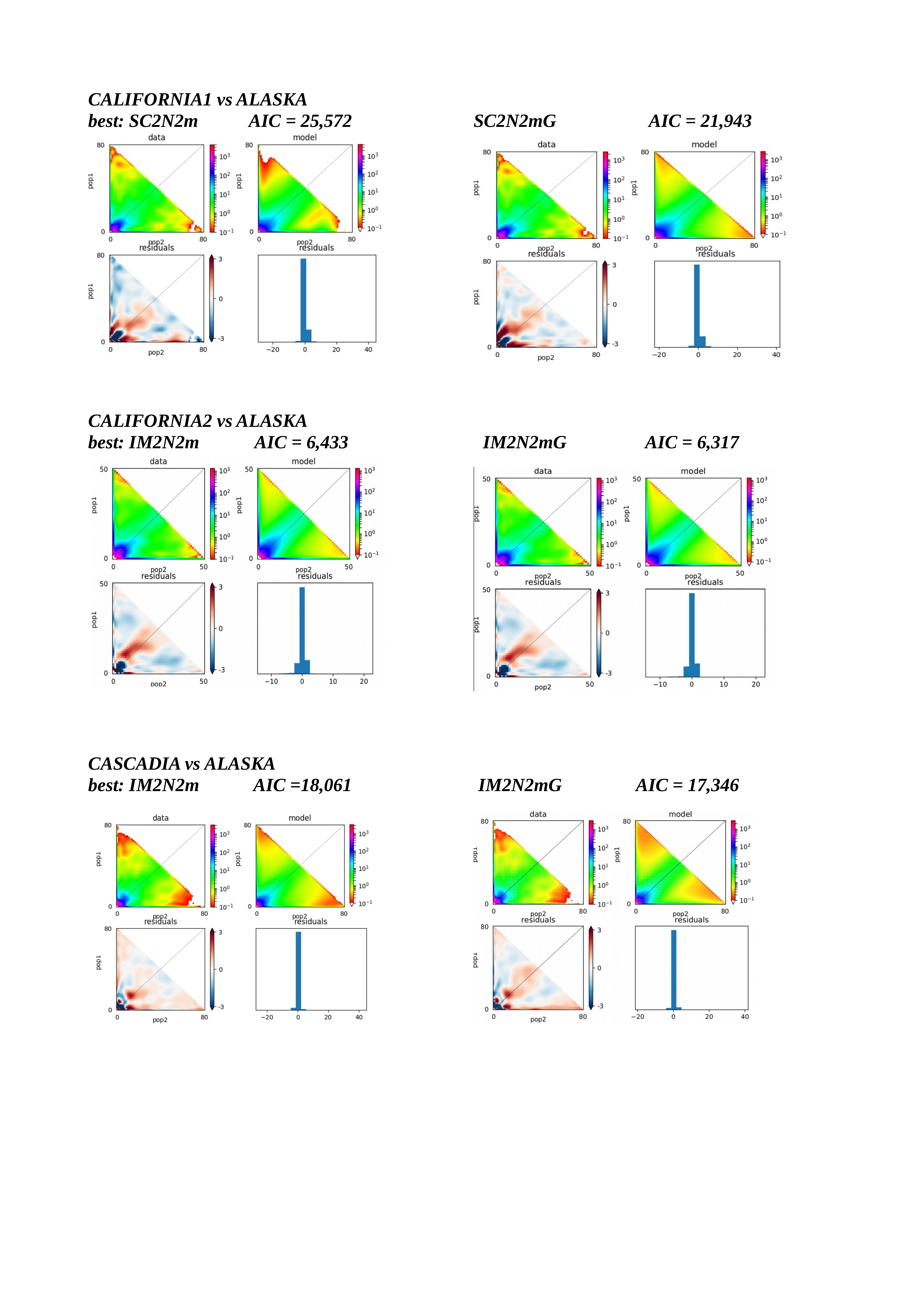

Supplement: S12 Fig — Each plot displays the observed jSFS (data), the modeled jSFS (model) and the residuals. Left part: Best model without population size change. Each best model was inferred using ΔAIC and AIC weights. Right panel: The same model as the left but including the possibility for population size change of the diverging daughter populations. (TIF) [file pgen.1008348.s012.tif]

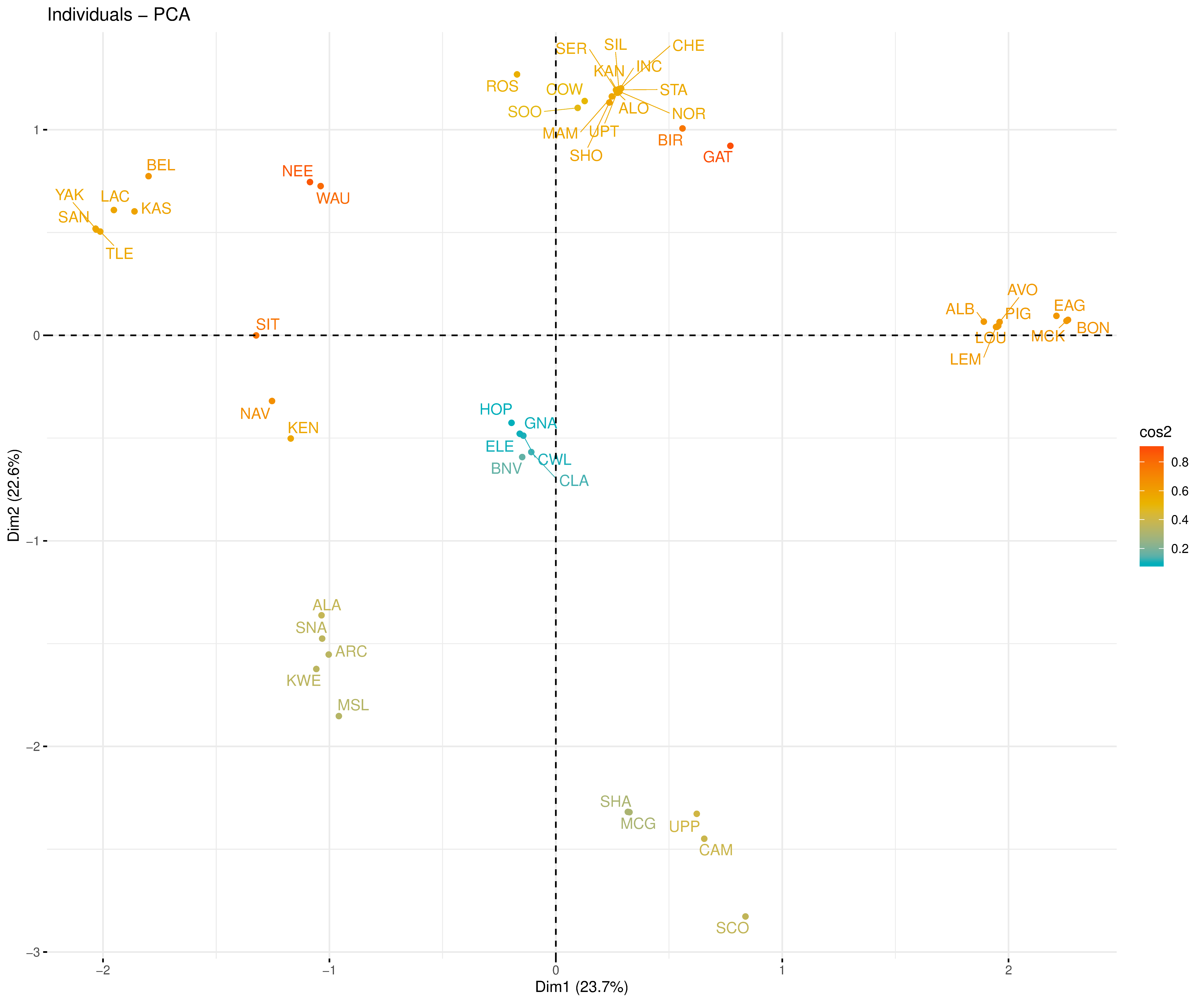

Supplement: S13 Fig — For easier interpretations of admixture coefficient among the 58 samples site a PCA was performed to summarized the distribution of admixture. Populations with lower cos2 contributed weakly to the plot and hence have higher admixture. (TIF) [file pgen.1008348.s013.tif]

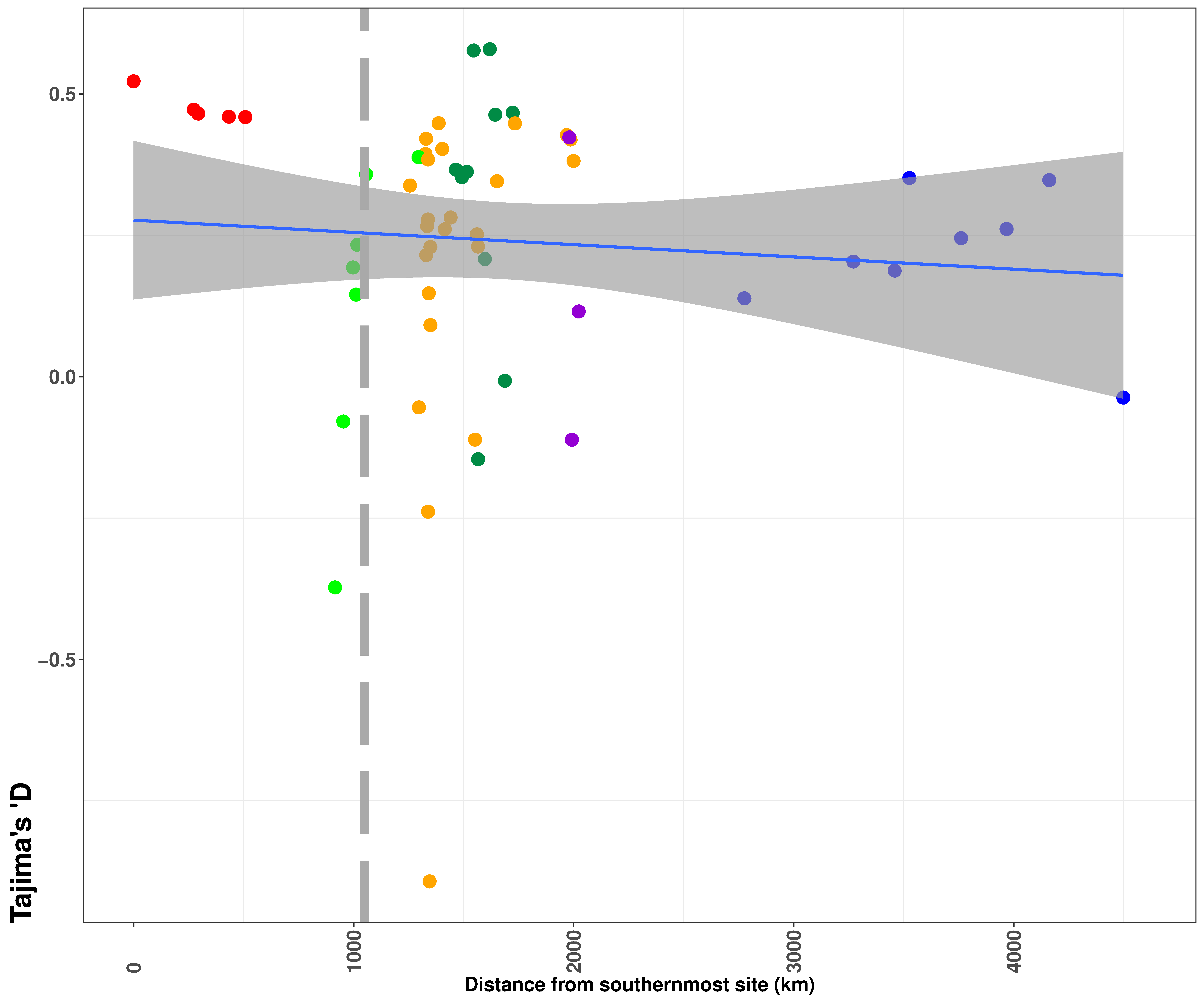

Supplement: S14 Fig — No significant relationship was observed (p = 0.5, r = -0.07), suggesting that each local population has undergone different evolutionary trajectory in post-glacial time. (TIF) [file pgen.1008348.s014.tif]

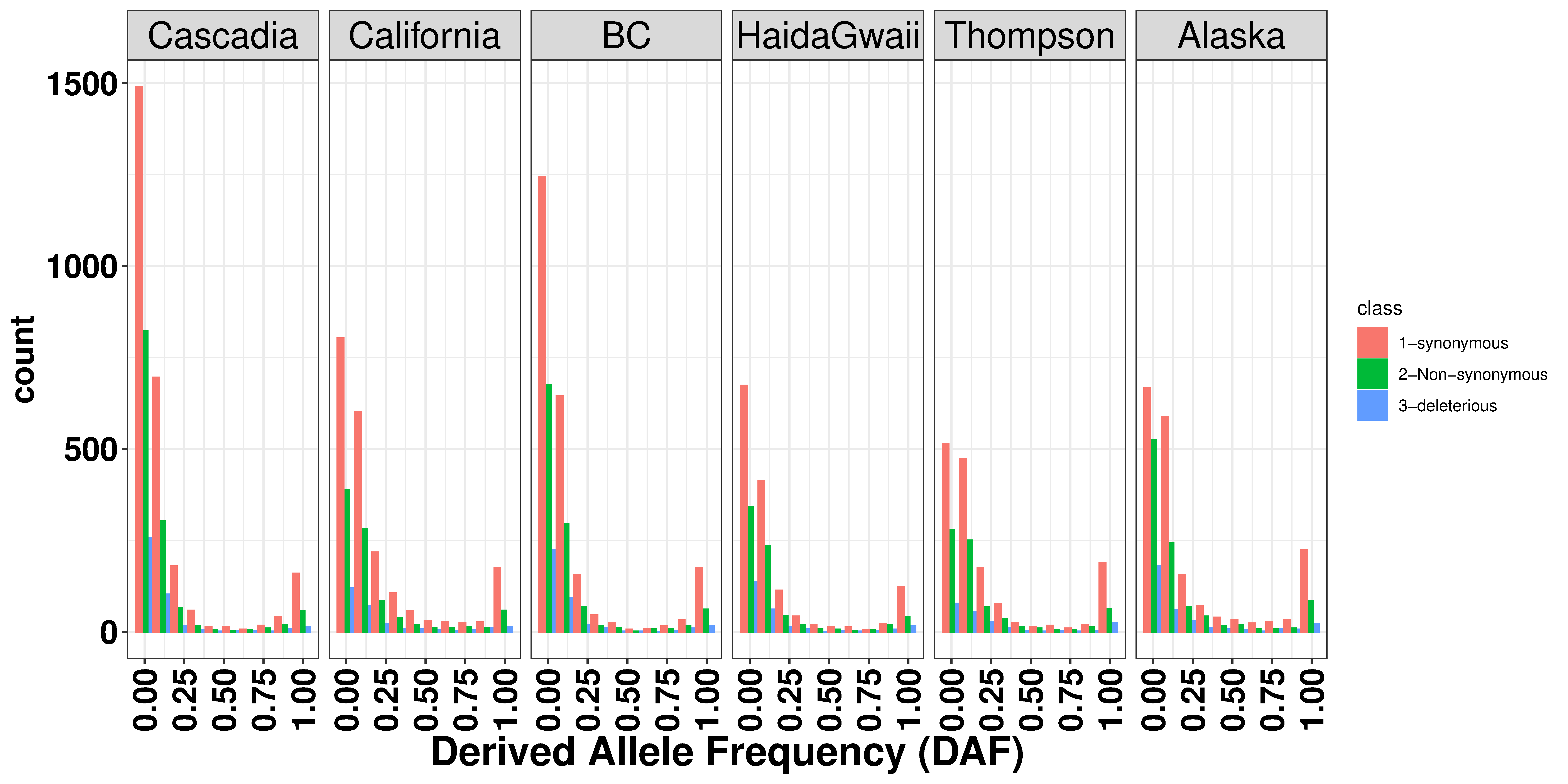

Supplement: S15 Fig — Data are normalized for a sample of size n = 102 corresponding to the smallest size for the combined samples in Haida Gwaii. (TIF) [file pgen.1008348.s015.tif]

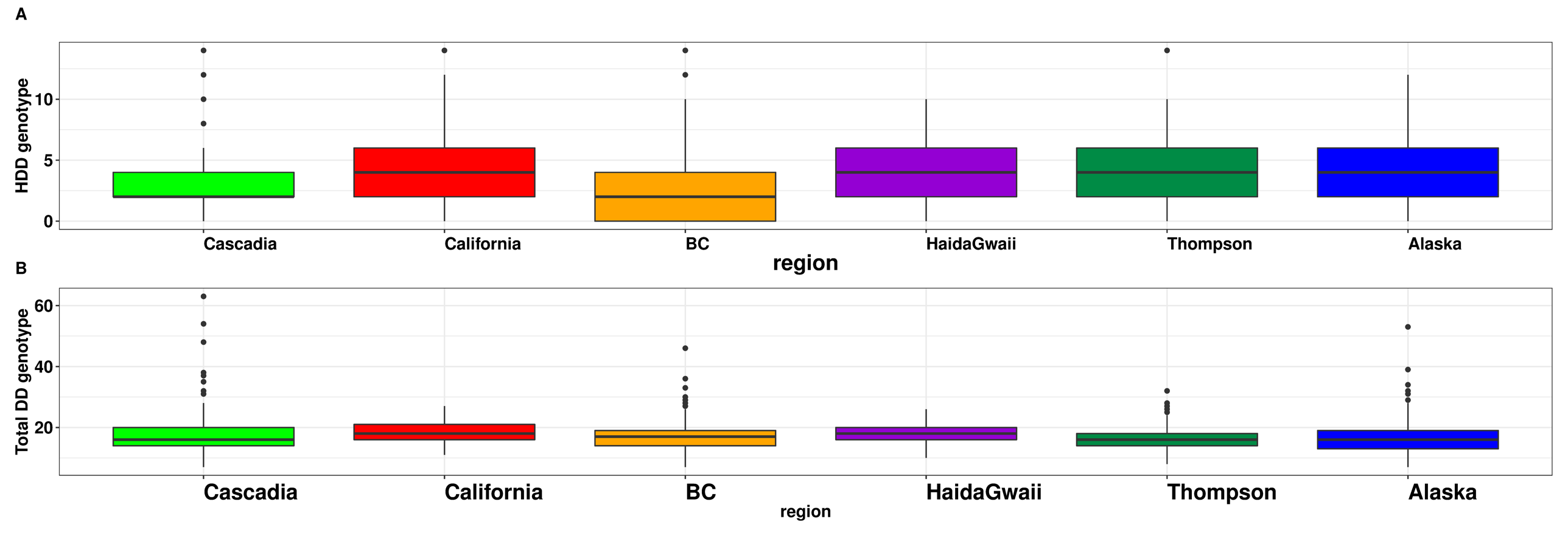

Supplement: S17 Fig: A) Distribution of the count of homozygous derived deleterious alleles in each major group. B) Distribution of the count of total derived deleterious alleles in each major group — (TIF) [file pgen.1008348.s017.tif]

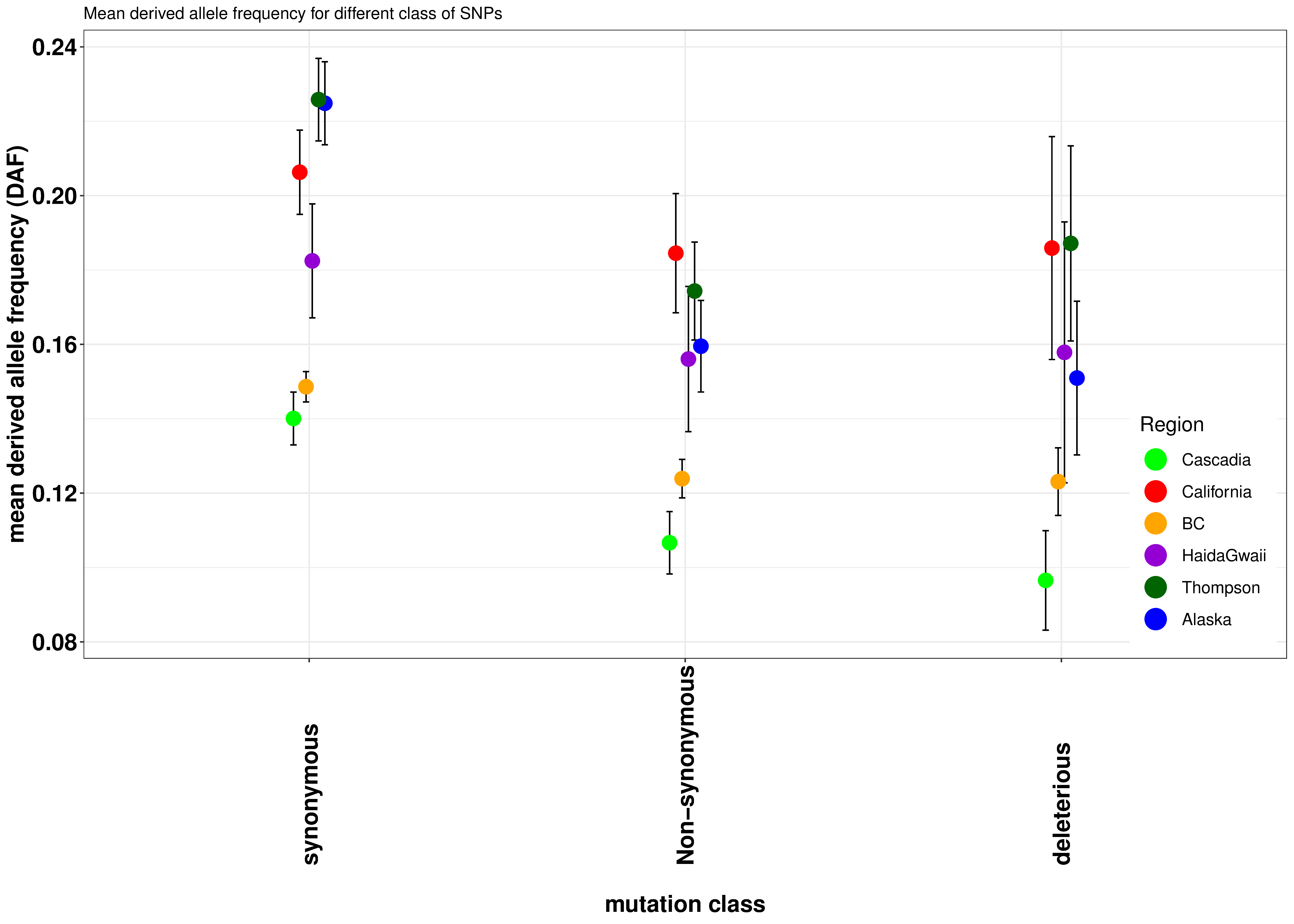

Supplement: S18 Fig — Displayed are the mean derived allele frequencies of polymorphic deleterious sites in each region +/- 2 standard deviation. (TIF) [file pgen.1008348.s018.tif]

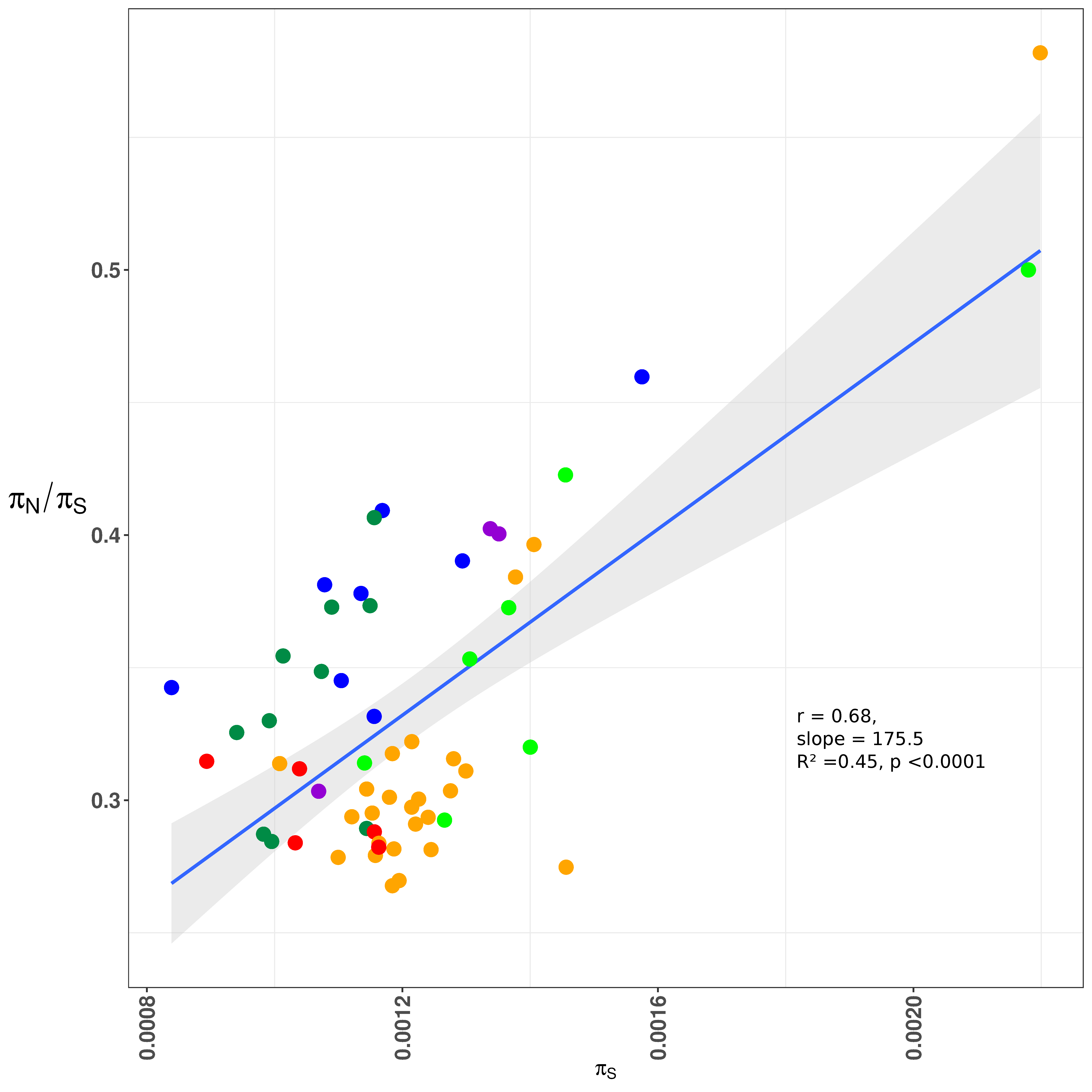

Supplement: S19 Fig — (TIF) [file pgen.1008348.s019.tif]

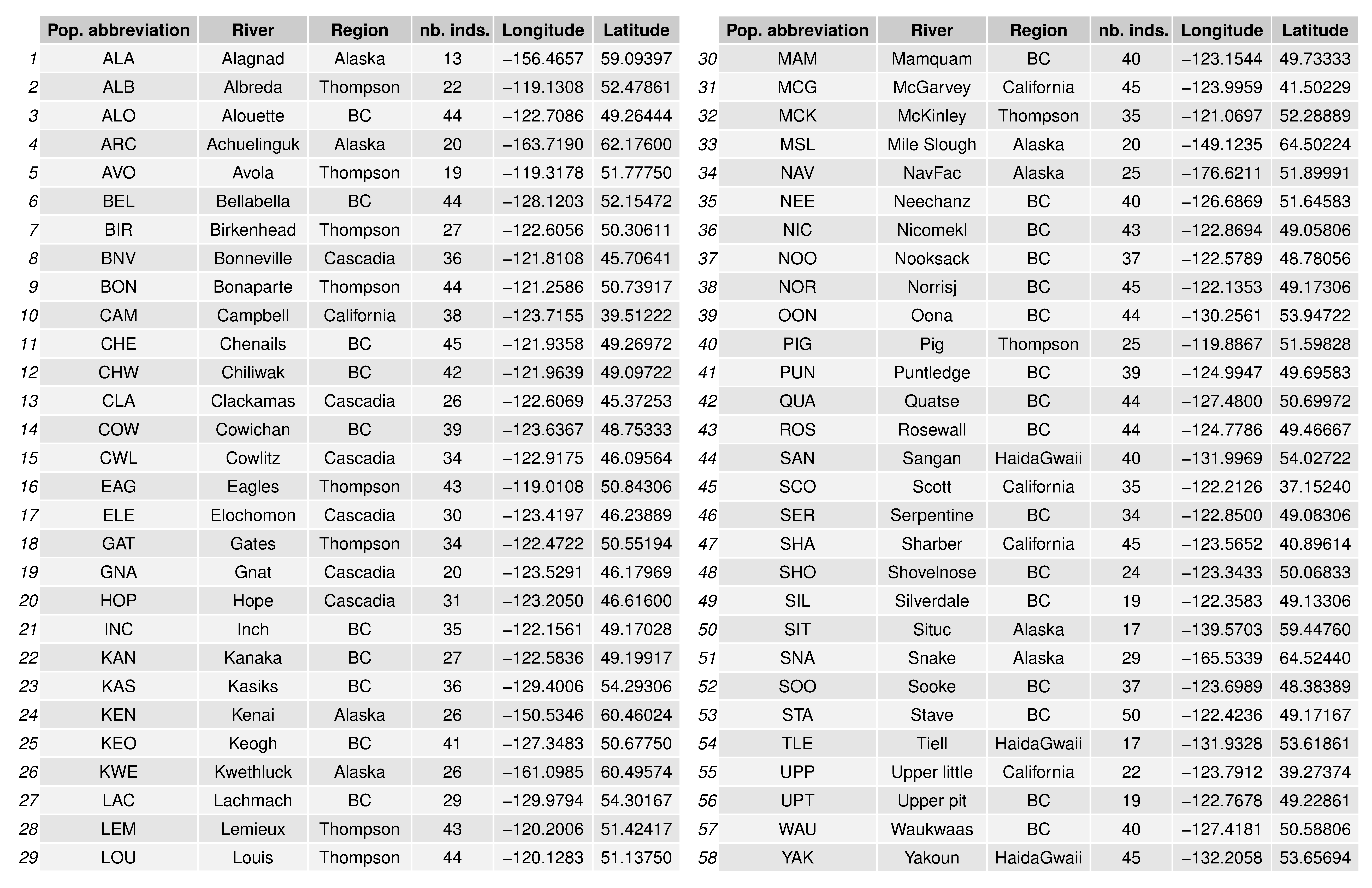

Supplement: S1 Table — Abbreviation, with corresponding river of sampling, Region and coordinates (Longitude and Latitude) of each sites used in the GBS data with the number of individuals provided (nb. Inds). (TIF) [file pgen.1008348.s020.tif]

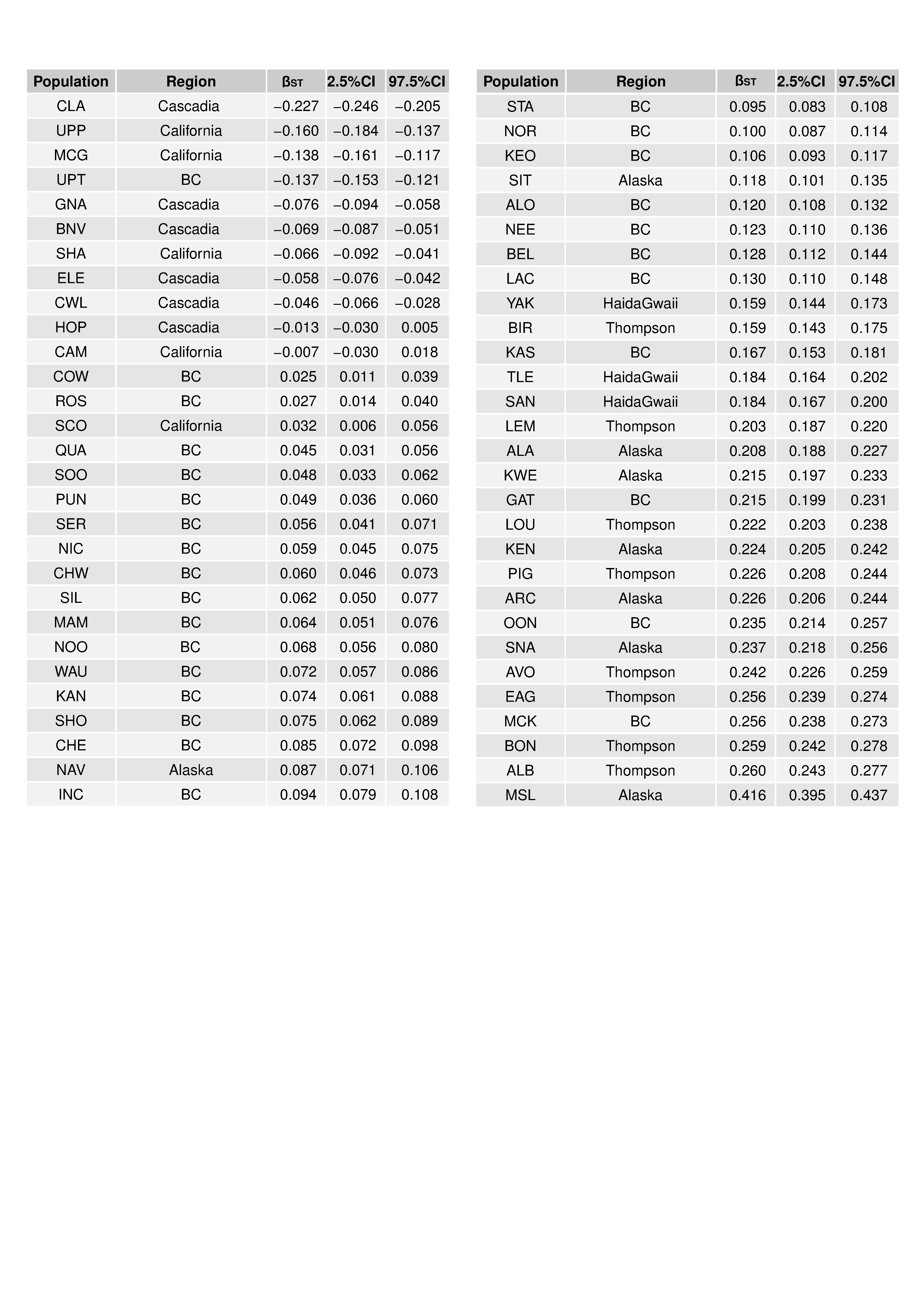

Supplement: S2 Table — 95% confidence intervals obtained after 1000 bootstraps. (TIF) [file pgen.1008348.s021.tif]

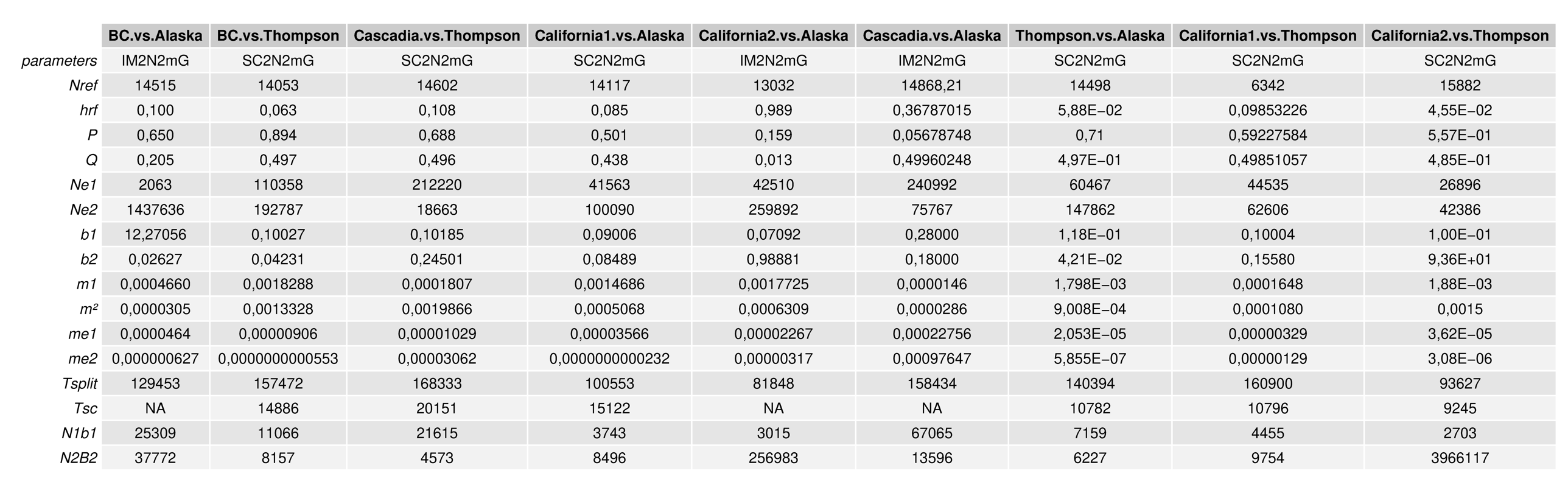

Supplement: S4 Table — Ne1 and Ne2, effective population size of the compared pair. m1 ← 2 and m2 ← 1, migration from population 2 to population 1 and migration from population 1 into population 2. me12 and me21, effective migration rate estimated in the most differentiated regions of the genome Ts: Split Time of the ancestral population in two population; Tsc: duration of the secondary contact P: proportion of the genome freely exchanged (1-P provides the proportion of the genome non-neutrally exchanged); Q: proportion of the genome with a reduced effective population size due to selection at linked sites; hrf = Hill-Robertson factor representing the reduction of Ne in the region Q with reduced Ne. (TIF) [file pgen.1008348.s023.tif]

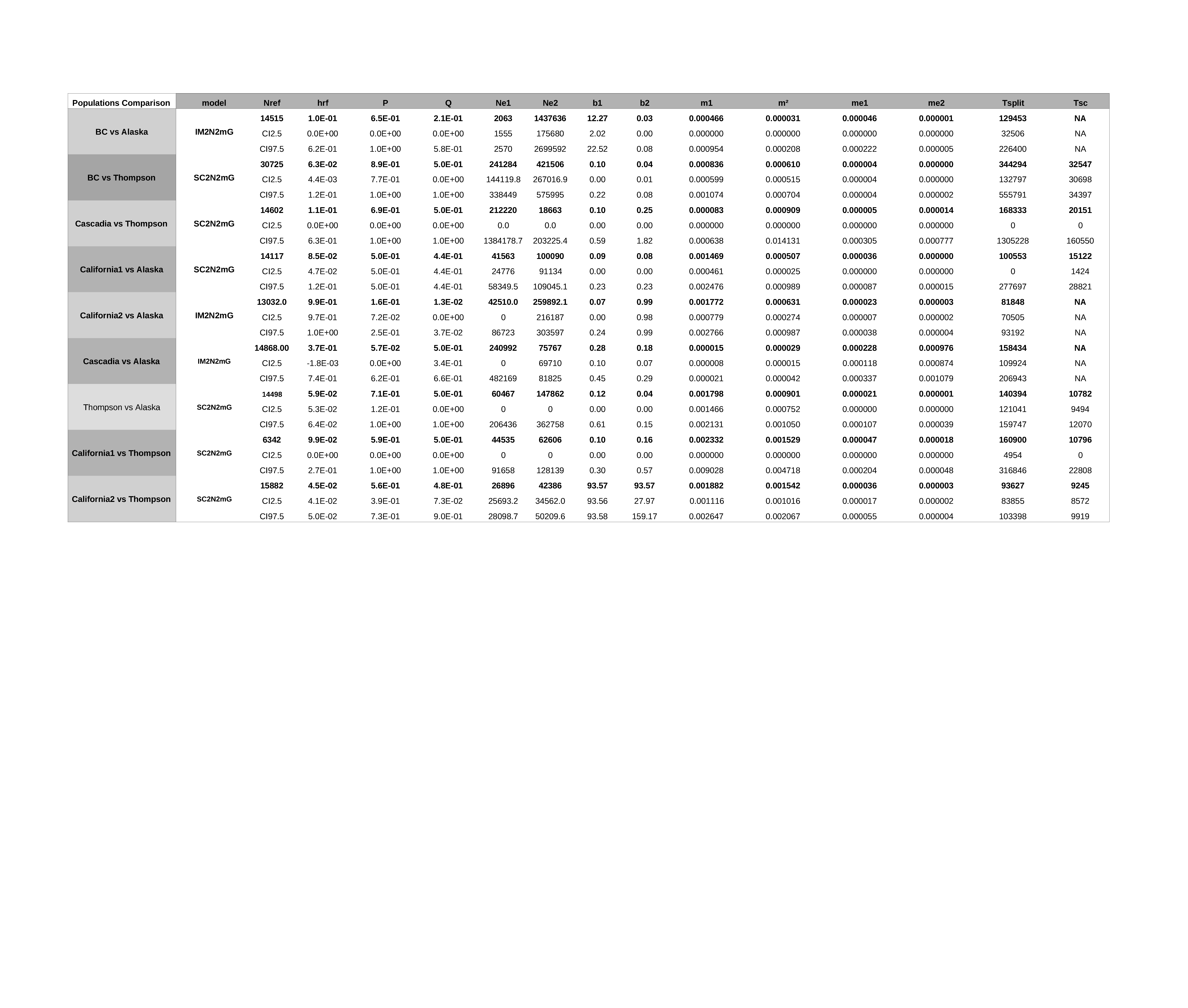

Supplement: S5 Table — (TIF) [file pgen.1008348.s024.tif]

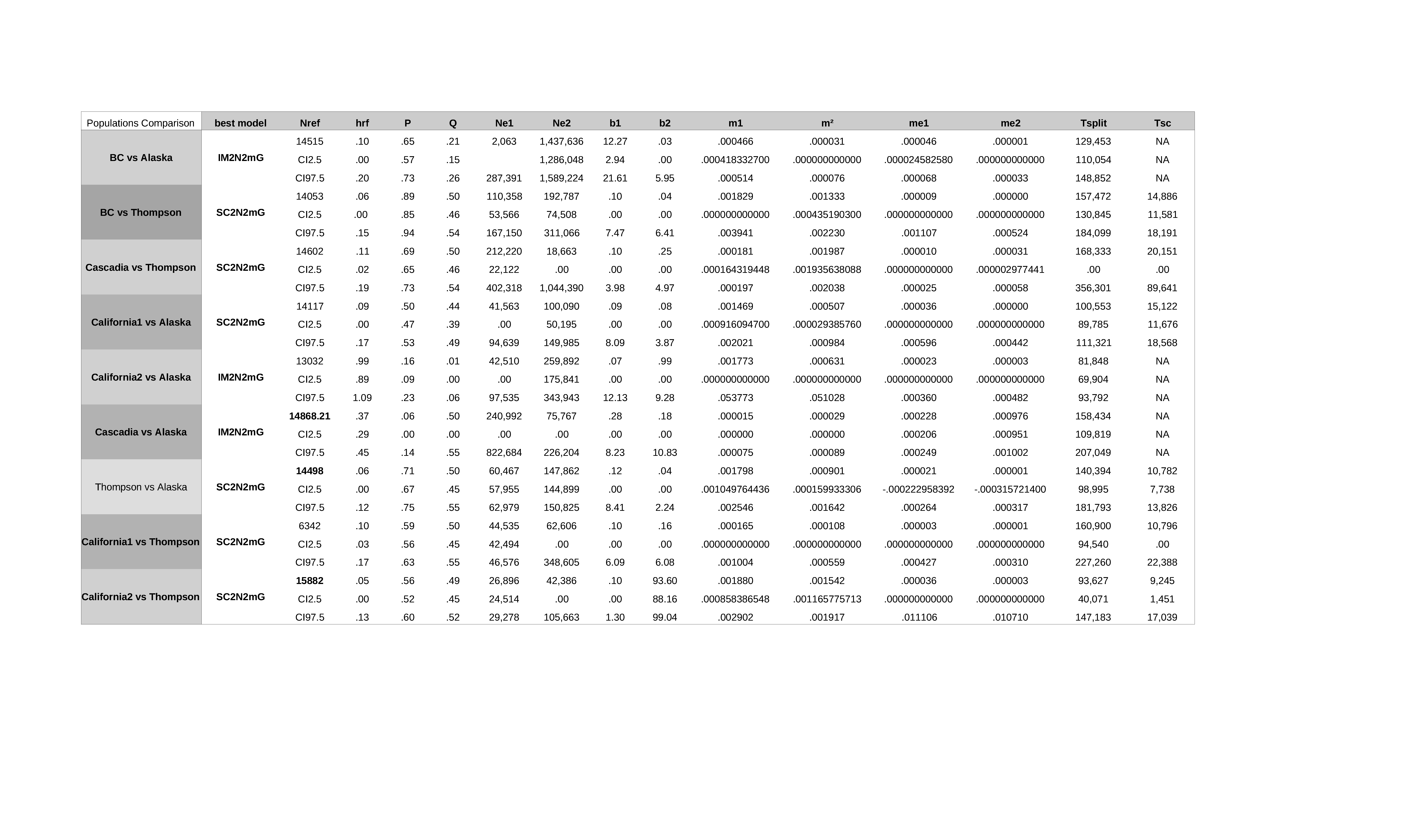

Supplement: S6 Table — (TIF) [file pgen.1008348.s025.tif]

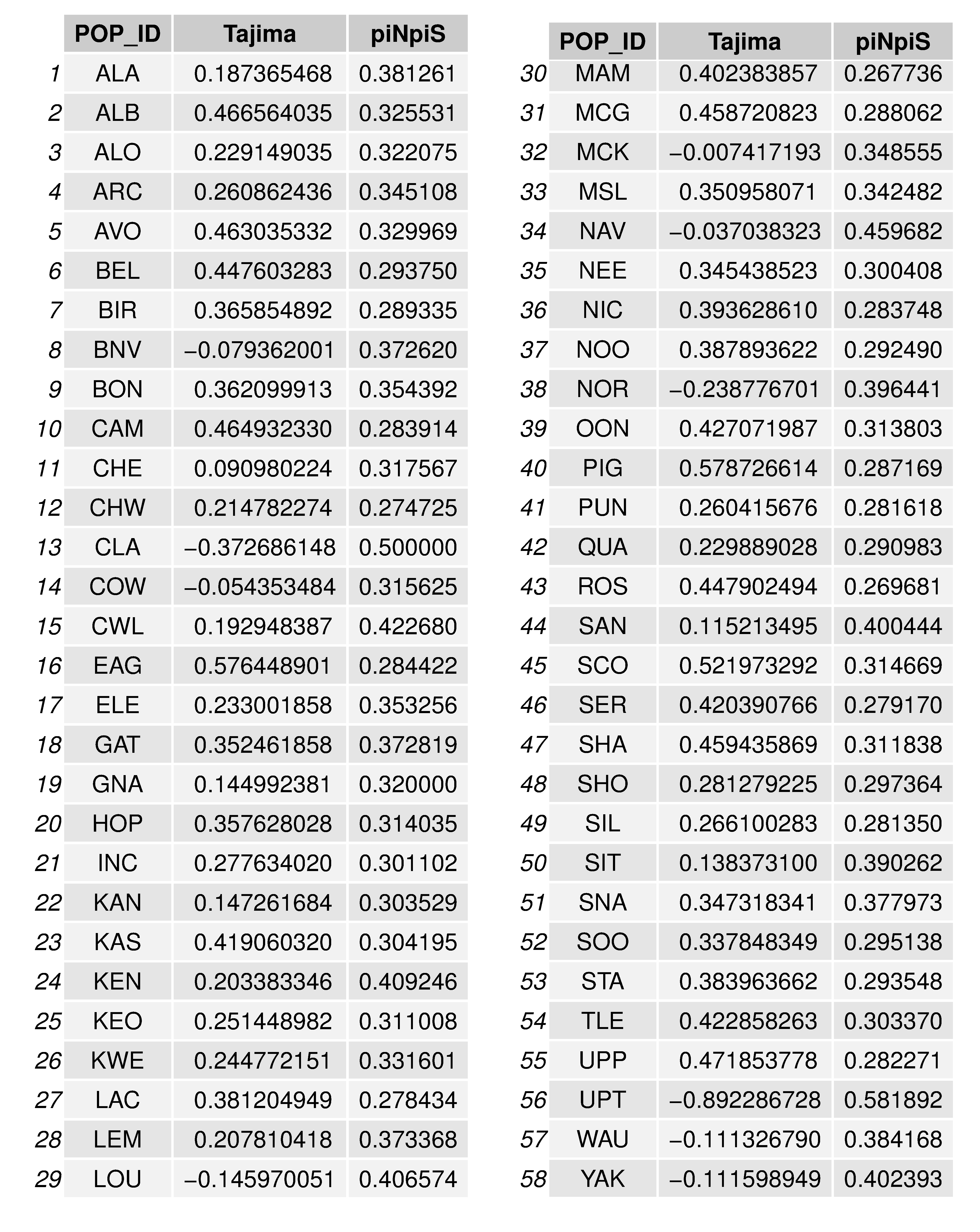

Supplement: S8 Table — (TIF) [file pgen.1008348.s027.tif]

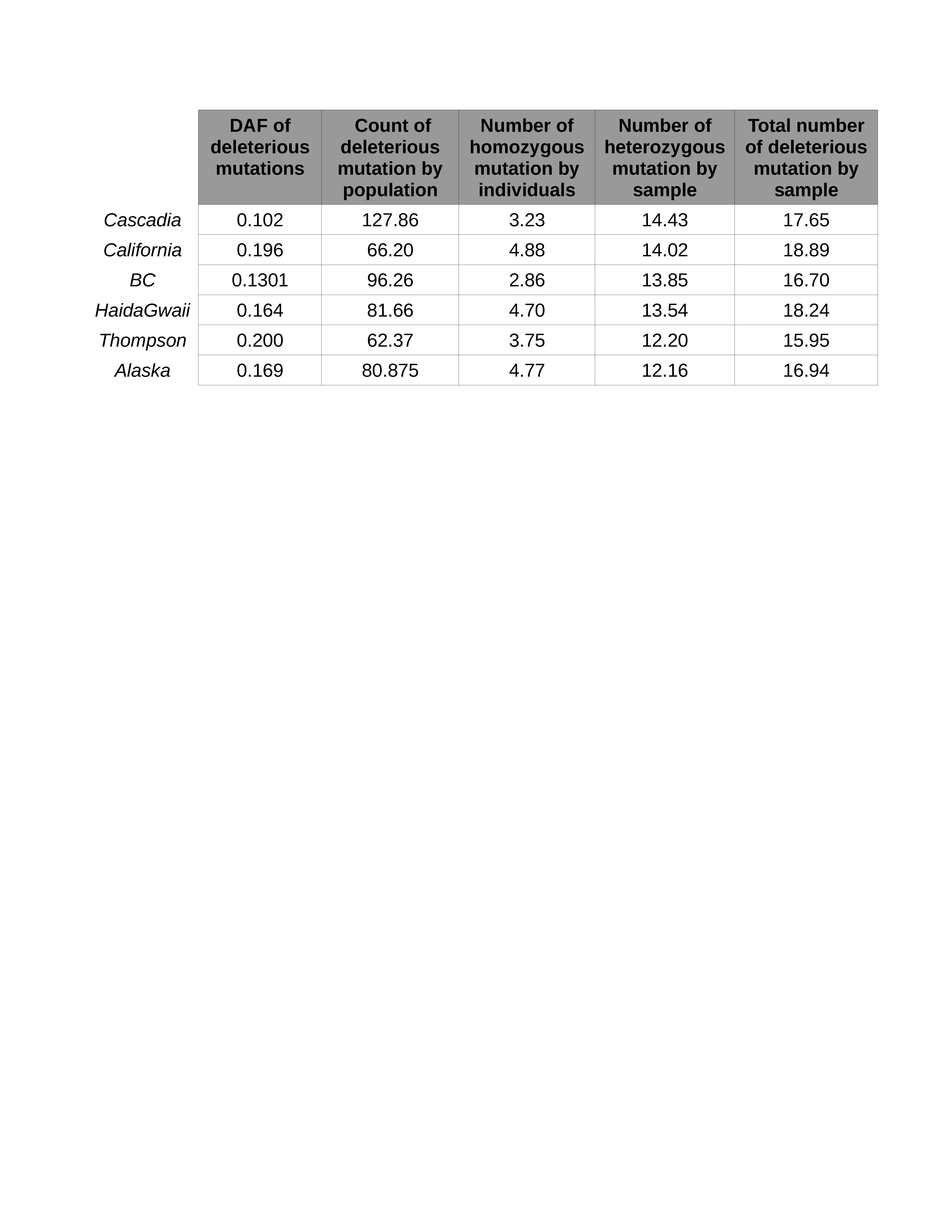

Supplement: S9 Table — 1)Derived Allele Frequency (DAF) of deleterious mutation, after averaging by rivers and then by major regional group. 2) Count of deleterious mutations in each river and then averaged by major regional group. 3) Number of homozygous derived deleterious mutations by individual, after averaging by rivers and then by major regional group. 4) Number of heterozygous mutations by individuals, after averaging by rivers and then by major regional group 5) Total load of derived deleterious mutations by individuals, after averaging by rivers and then by major regional group. (TIF) [file pgen.1008348.s028.tif]

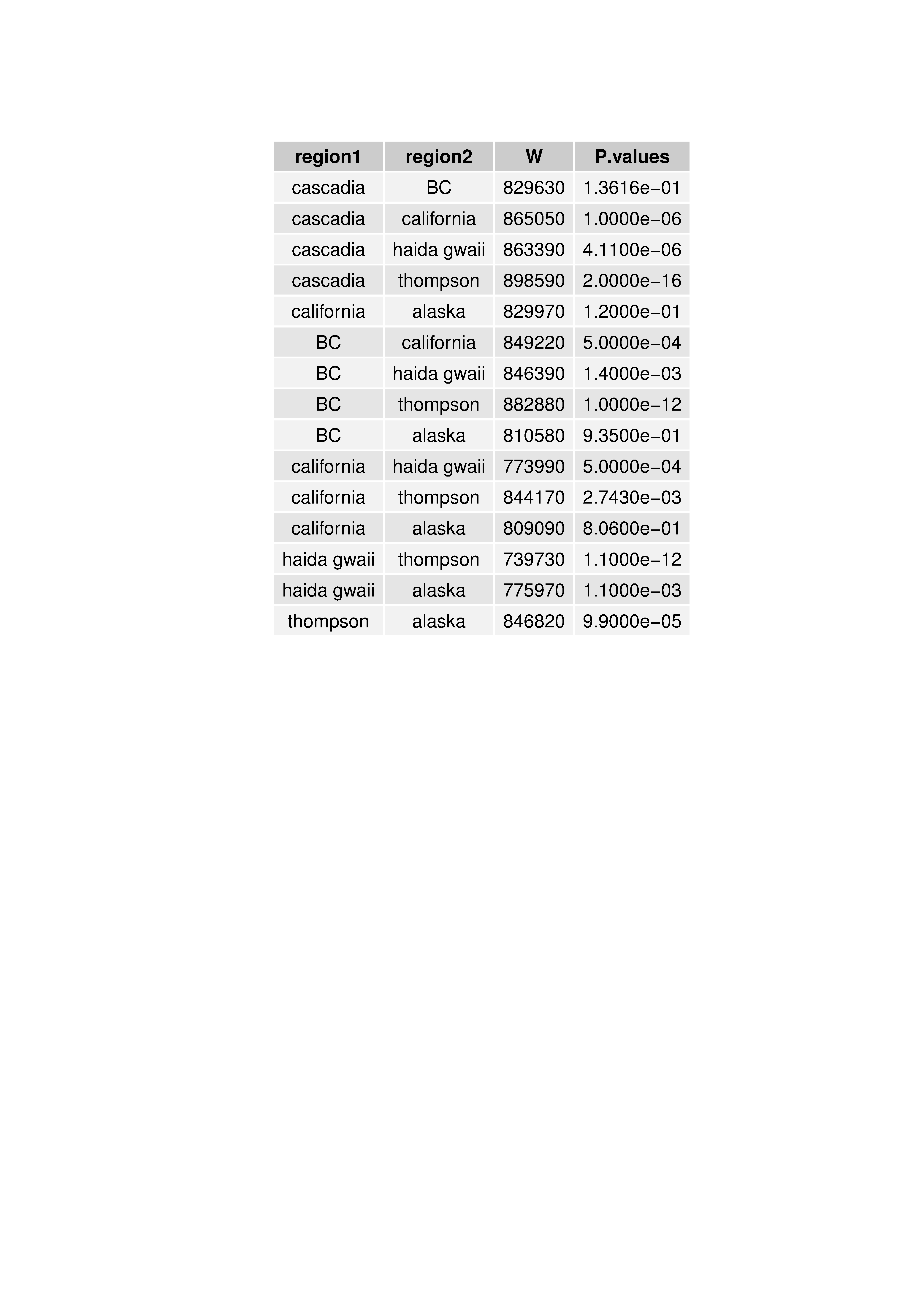

Supplement: S10 Table — (TIF) [file pgen.1008348.s029.tif]

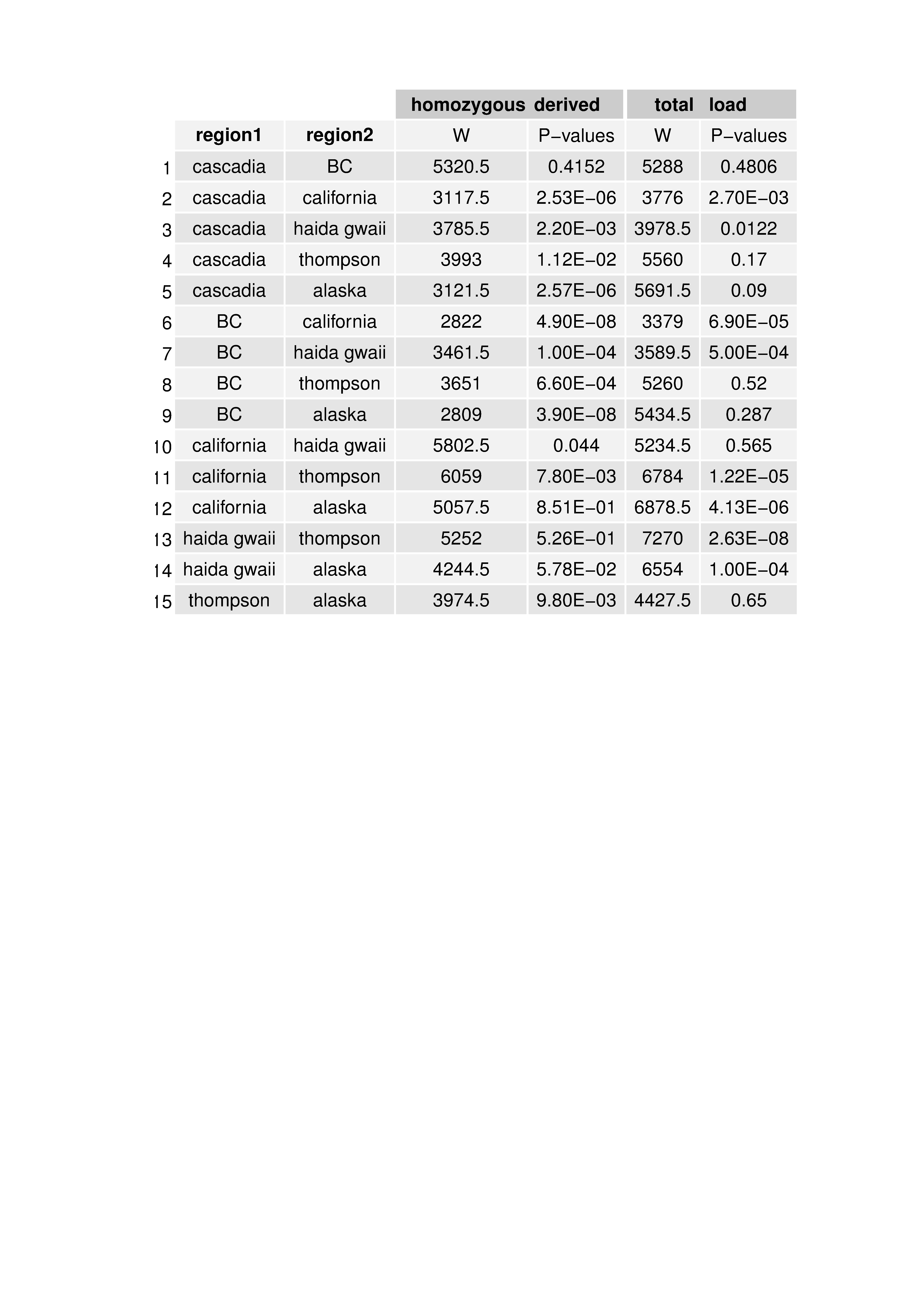

Supplement: S11 Table — (TIF) [file pgen.1008348.s030.tif]
